# Ambra1 Shapes Hippocampal Inhibition/Excitation Balance: Role in Neurodevelopmental Disorders

**DOI:** 10.1007/s12035-018-0911-5

**Published:** 2018-02-27

**Authors:** Annalisa Nobili, Paraskevi Krashia, Alberto Cordella, Livia La Barbera, Maria Concetta Dell’Acqua, Angela Caruso, Annabella Pignataro, Ramona Marino, Francesca Sciarra, Filippo Biamonte, Maria Luisa Scattoni, Martine Ammassari-Teule, Francesco Cecconi, Nicola Berretta, Flavio Keller, Nicola Biagio Mercuri, Marcello D’Amelio

**Affiliations:** 1 Department of Experimental Neurosciences, IRCCS Santa Lucia Foundation, 00143 Rome, Italy; 2 Department of Medicine, University Campus-Biomedico, 00128 Rome, Italy; 3 Department of Systems Medicine, University of Rome ‘Tor Vergata’, 00133 Rome, Italy; 4 Research Coordination and Support Service, Istituto Superiore di Sanità (ISS), 00161 Rome, Italy; 5 Institute of Cell Biology and Neurobiology (IBCN), National Research Council (CNR), 00143 Rome, Italy; 6 Institute of Histology and Embryology, “A. Gemelli” Faculty of Medicine, Catholic University of the Sacred Heart, 00168 Rome, Italy; 7 Department of Biology, University of Rome ‘Tor Vergata’, 00133 Rome, Italy; 8 Cell Stress and Survival Group, Danish Cancer Society Research Center, DK-2100 Copenhagen, Denmark; 9 Department of Pediatric Hematology and Oncology, IRCSS Bambino Gesu Children’s Hospital, 00165 Rome, Italy

**Keywords:** Ambra1, Neurodevelopmental disorders, Parvalbumin interneurons, Hippocampus, Inhibition/excitation ratio, Synaptic plasticity

## Abstract

Imbalances between excitatory and inhibitory synaptic transmission cause brain network dysfunction and are central to the pathogenesis of neurodevelopmental disorders. Parvalbumin interneurons are highly implicated in this imbalance. Here, we probed the social behavior and hippocampal function of mice carrying a haploinsufficiency for *Ambra1*, a pro-autophagic gene crucial for brain development. We show that heterozygous Ambra1 mice (Ambra^+/−^) are characterized by loss of hippocampal parvalbumin interneurons, decreases in the inhibition/excitation ratio, and altered social behaviors that are solely restricted to the female gender. Loss of parvalbumin interneurons in Ambra1^+/−^ females is further linked to reductions of the inhibitory drive onto principal neurons and alterations in network oscillatory activity, CA1 synaptic plasticity, and pyramidal neuron spine density. Parvalbumin interneuron loss is underlined by increased apoptosis during the embryonic development of progenitor neurons in the medial ganglionic eminence. Together, these findings identify an Ambra1-dependent mechanism that drives inhibition/excitation imbalance in the hippocampus, contributing to abnormal brain activity reminiscent of neurodevelopmental disorders.

**Electronic supplementary material:**

The online version of this article (10.1007/s12035-018-0911-5) contains supplementary material, which is available to authorized users.

## Introduction

Neurodevelopmental disorders including autism and schizophrenia are disease spectrums with considerable overlapping phenotypes, including deficits in cognition and complex information processing, and behaviors such as anxiety and impaired social interaction [1–6]. Genetic studies have made enormous progress in identifying chromosomal regions related with autism-spectrum disorders (ASDs) and schizophrenia [7–9]. Multiple lines of evidence implicate alterations in the ratio between neuronal inhibition and excitation as a common pathophysiological mechanism [10–12]. Notably, these alterations appear to affect the development, number, or function of parvalbumin (PV)-expressing GABAergic interneurons [13–16], resulting in reduced inhibition of cortical and hippocampal pyramidal neurons and impairments in the synchronization of neuronal networks [17].

The *Ambra1* gene encodes the activating molecule in Beclin1-regulated autophagy (Ambra1; [18]), a large protein which interacts with Beclin1, a component of the class III phosphatidylinositol 3-kinase/Vps34 complex regulating autophagosome formation in mammals [19]. Ambra1 is produced during embryogenesis and is crucial for brain development. In fact, homozygous Ambra1^−/−^ mouse embryos show serious brain malformations, resulting in embryonic death at embryonic day 10 (e10)-e14.5 [18]. Postnatally, Ambra1 is expressed in the cerebellum and striatum, as well as in the cortex and hippocampus where it is mainly immunolocalized in neurons, with little or no expression in glia [18, 20].

In line with the importance of Ambra1 for normal brain development, a genome-wide association study on schizophrenia patients identified a genetic risk variation in a region on chromosome 11 (11p11.2) containing the *AMBRA1* gene [21]. Risk allele-carriers showed a higher blood oxygen level-dependent response in the prefrontal cortex during a flanker task, indicating that the risk allele is associated with impulsive-related traits [21, 22]_._ Notably, a behavioral study on heterozygous Ambra1 mice (Ambra1^+/−^) revealed an autism-like phenotype that was restricted only to females [23] and a recent work proved an association between autism and intronic single nucleotide polymorphisms of the *AMBRA1* gene in human female patients [24].

Despite its implications in both schizophrenia and ASDs, the underlying neurophysiological mechanism linking Ambra1 loss to these pathologies is unknown. Here, we show that haploinsufficiency for the *Ambra1* gene results in a reduced number of PV interneurons in the Ambra1^+/−^ mouse hippocampus, resulting in alterations in inhibitory synaptic transmission, network oscillations, and synaptic plasticity. These deficits are restricted to the female gender and are accompanied by social interaction impairments. Our data show an increased susceptibility of female Ambra1^+/−^ PV interneuron progenitors to apoptosis in the medial ganglionic eminence (MGE), providing new insights into the importance of Ambra1 for the genesis and proper interneuron development.

## Materials and Methods

### Animals

All experiments complied with the ethical guidelines of the EU Directive 2010/63EU and the Italian Health Ministry (Art.31, D.Lgs 26/2014; protocol #34/2014-PR). Heterozygous male and female Ambra1^+/−^ [18] and wild-type (WT) mice were used at 2–3 months of age and at e10.5 and e13.5. Heterozygous Ambra1^+/−^ mice were obtained by breeding Ambra1^+/−^ males with C57BL/6J females. Animals were housed in a temperature- and humidity-controlled environment (free access to food and water; 12 h dark/light cycle).

### Genotyping

To determine genotype and sex, DNA was isolated from tails digesting the tissues at 56 °C in lysis buffer containing (in mM): 50 Tris-HCl pH 8.0, 100 EDTA pH 8.0, 100 NaCl, 1% SDS, and 1 μg/μl proteinase K (nzytech). DNA was purified by extraction with Phenol:Chloroform. Primers were synthesized by Eurofins.

For genotyping, *Ambra1* primer sequences are: 5′-AGACAATCGGCTGCTCTGAT-3′; 5′-ATACTTTCTCGGCAGGAGCA-3′. DNA amplification was performed according to the following program: 1 cycle at 95 °C (4 min), 25 cycles at 95 °C (1 min) then 56 °C (30 s) and 72 °C (30 s), and 1 cycle at 72 °C (10 min).

For determining the sex of embryos *Sry* primer sequences are: 5′-TCATGAGACTGCCAACCACAG-3′; 5′-CATGACCACCACCACCACCAA-3′; *Myog* primer sequences are 5′-TTACGTCCATCGTGGACAGC-3′, 5′-TGGGCTGGGTGTTAGTCTTA-3′; DNA amplification was performed according to the following program: 1 cycle at 94 °C (5 min), 35 cycles at 94 °C (1 min) then 58 °C (45 s) and 72 °C (1 min), and 1 cycle at 72 °C (10 min).

PCR products were electrophoresed on 1.5% agarose gels and visualized with GelRed Nucleic Acid Gel Stain (Biotium) under UV light with the Kodak EDAS 290 imaging system.

### Social Interaction

For the female-female social interaction test, an unfamiliar female WT mouse (C57BL/6J), matched to the test female mouse by age and body weight, was placed into the standard home-cage of the isolated test subject and ultrasonic vocalization emission and behaviors of each test mouse were recorded for a 3-min test session, as previously described [25]. For the male-female social interaction test, male test mice were evaluated with a C57BL/6J female in estrous. On the testing day, the vaginal estrous condition of each unfamiliar C57BL/6J female was assessed. Only females in estrous were selected for this test and each mouse was used only twice. For the female-female and the male-female social interaction tests, in addition to the test animal and unfamiliar C57BL/6J mice, the cage contained litter and the lid was removed. C57BL/6J mice were maintained in social groups of four per cage. Each unfamiliar C57BL/6J mouse matched the test mouse by age and body weight.

Audio recordings were used for quantitative analysis of ultrasonic vocalization numbers, as previously described [26]. Briefly, the ultrasonic microphone (Avisoft UltraSoundGate condenser microphone capsule CM16; Avisoft Bioacoustics, Germany) was mounted 20 cm above the cage and the ultrasonic vocalizations were recorded using the Avisoft RECORDER software (version 3.2). Settings included sampling rate at 250 kHz; format 16 bit. The ultrasonic microphone was sensitive to frequencies between 10 and 180 kHz. For acoustical analysis, recordings were transferred to Avisoft SASLabPro (version 4.40) and a fast Fourier transformation (FFT) was conducted. Spectrograms were generated with FFT-length of 512 points and a time window overlap of 75% (100% Frame, Hamming window). The spectrogram was produced at a frequency resolution of 488 Hz and a time resolution of 1 ms. A lower, 20 kHz, cut-off frequency was used to reduce background noise outside the relevant frequency band to 0 dB. For audio recording analysis, it is possible to differentiate which mouse is vocalizing during the tasks: in the male-female context, the male socially investigates the female and simultaneously vocalizes to elicit social proximity and check her sexual receptivity [27–29]; in the female-female context, only the resident female is vocalizing and socially investigating the intruder female to enhance social proximity [30–32].

For video recordings (with a Sony monochrome charge-coupled videocamera mounted facing the cage side), scoring of sociability was conducted with the Noldus Observer 10XT software (Noldus Information Technology; Leesburg, VA, USA). Sociability was scored from video recordings, for the duration of the social interaction (sum of anogenital/body/head sniffing area). Observations of mounting, fighting, tail rattling, and wrestling were excluded.

Start times for the audio and video files were synchronized. However, because vocalizations occurred in a time-frame of milliseconds and behavioral events occurred in a time-frame of seconds, it was not possible to synchronize scoring of vocalizations with behaviors using the currently available recording technology. The software used for spectrographic (Avisoft Bioacoustics, Avisoft SASLabPro v4.40) and behavioral (Noldus, Observer 3.0) analysis cannot be combined on the same screen because they proceed with different speeds. Scoring of audio and video files was conducted by an investigator blind to the mouse genotype. Experiments were performed during light cycle.

### Three-Chamber Task

Social approach was tested using a three-chambered apparatus [33], consists of a rectangular box (60 cm length × 40 cm width × 40 cm height) of clear Plexiglas, divided into a central chamber and two side chambers. Retractable doors (6 cm width × 4 cm height) allowed access to the side chambers. A top-mounted camera was positioned directly above the apparatus to record the sessions.

The test mouse was first acclimated to the apparatus before testing, beginning with a 10-min habituation session in the empty central chamber, followed by a second 10-min session in which the mouse was allowed to explore all three empty chambers. The second habituation session served to confirm a lack of innate side-chamber preference. Following habituation, the test mouse was confined to the central chamber, an object (an inverted wire pencil cup) was placed in one side-chamber, and a stranger mouse, enclosed in an inverted wire pencil cup, was placed in the other side-chamber. The side-doors were simultaneously lifted and the test mouse could choose between spending time in the side with the stranger mouse versus the object (sociability phase, 10 min). Following sociability phase, each mouse was tested for social novelty (10 min) to evaluate social preference for a second stranger mouse (non-familiar mouse), enclosed in the previously empty cup. The test mouse could choose between the familiar and the non-familiar mouse. For the sociability and social novelty phase, the animals serving as strangers were C57BL/6J mice that had previously been habituated to placement in the small cage and had no prior contact with the subject. Stranger mice, matched to the test mice by age and sex, were enclosed in the wire cup to ensure that all social approach was initiated by the test mouse, and to avoid aggressive and sexual behaviors toward test mice, while allowing visual, olfactory, auditory, and partial tactile contact through the wire bars. A weighted cup was placed on the top of each inverted cup to prevent climbing by the test mouse. The location (left or right) of the object and the stranger mice was systematically alternated across subjects. The apparatus was cleaned with 70% ethanol and water between experiments. Scoring was performed using Noldus Observer XT event coding software (Noldus Information Technology; Leesburg, VA, USA). Experiments were performed during light cycle. Number of chamber entries, time spent in each chamber, and cumulative time spent sniffing the object and the stranger mouse were later scored from video recordings, by a researcher uninformed of genotype. An entry was defined as all four paws in one chamber; direction of the head, facing toward the cup, defined sniffing.

### Protein Extraction

Hippocampi were isolated from the entire brain. For GABAergic neuron marker analysis, tissues were homogenized in lysis buffer containing (in mM) 320 sucrose, 50 NaCl, 50 Tris-HCl pH 7.5, 1% Triton X-100, 1 sodium orthovanadate, 5 β-glycerophosphate, 5 NaF, and protease inhibitor cocktail. For membrane protein analysis (receptor subunits and structural proteins), tissues were homogenized in RIPA containing (in mM) 50 Tris-HCl pH 7.4, 150 NaCl, 1 EDTA, 5 MgCl_2_, 1% Triton X-100, 0.25% Na-deoxycholate, 0.1% SDS, and protease inhibitor cocktail. For embryos, tissues were homogenized in lysis buffer containing 10% glycerol. Homogenates were incubated on ice (30 min) and centrifuged at 15,000 *g* at 4 °C (10 min). The total protein content of the supernatant was determined by the Bradford method.

### Immunoblotting

Proteins were applied to SDS-PAGE and electroblotted on a polyvinylidene difluoride membrane. Immunoblotting analysis was performed using a chemiluminescence detection kit. The relative immunoreactivity levels were determined by densitometry using ImageJ (https://imagej.nih.gov/). Membranes were stripped using Re-Blot Plus Strong Solution (Millipore) for 15 min at room temperature.

Primary antibodies: Ambra1 (1:1000; Millipore, ABC131, RRID:AB_2636939), GAD67 (1:1000; Abcam, ab97739, RRID:AB_10681171), GAD65 (1:1000; Abcam, ab26113, RRID:AB_448989), VGAT (1:1000; Santa Cruz, sc-49,574, RRID:AB_2189931), Calretinin (1:1000; Millipore, MAB1568, RRID:AB_1674238), Calbindin (1:1000; Sigma-Aldrich, C9848, RRID:AB_476894), PV (1:1000; Abcam, ab11427, RRID:AB_298032), PSD95 (1:1000; Millipore, MAB1598, RRID:AB_1715818), GluN1 (1:1000; Santa Cruz, sc-1467, RRID:AB_670215), GluN2A (1:250; Santa Cruz, sc-1468, RRID:AB_670223), GluN2B (1:1000; Millipore, 06-600, RRID:AB_310193), P-GluA1 Ser845 (1:1000; Millipore, AB5849, RRID:AB_92079), GluA1 (1:1,000; Millipore, 04-855, RRID:AB_1977216), VGLUT1 (1:1000; Millipore, MAB5502, RRID:AB_262185), Actin (1:25,000; Sigma-Aldrich, A5060, RRID:AB_476738).

Secondary antibodies: goat anti-mouse IgG (1:3000; Bio-Rad), goat anti-rabbit IgG (1:3000; Bio-Rad), rabbit anti-goat IgG (1:3000; Bio-Rad). Membranes were stripped using Re-Blot Plus Strong Solution (Millipore) for 15 min at room temperature.

### Immunohistochemistry/Immunofluorescence

Adult mice were anesthetized with Rompun (20 mg/ml, 0.5 ml/kg, i.p., Bayer) and Zoletil (100 mg/ml, 0.5 ml/kg, Virbac) and perfused transcardially with 50 ml saline followed by 50 ml of 4% paraformaldehyde in phosphate buffer saline (PBS; 0.1 M, pH 7.4), as described in [34]. Brains were removed, post-fixed for 4 h, and transferred to 30% sucrose in PBS at 4 °C until they sank. Brains were cut coronally at 30 μm with a cryostat and dorsal hippocampal slices (A/P − 1.7/− 2.5; [35]) were collected. Embryo were fixed in 4% paraformaldehyde in PBS overnight and transferred to 30% sucrose in PBS at 4 °C until they sank. Brains were cut coronally at 40 μm with a cryostat and the slices were collected.

For immunohistochemistry or Ambra1/PV staining, every second slice was processed alternatively. To analyze PV cells, the slices selected for immunohistochemistry were processed with an anti-PV polyclonal antibody by the avidin-biotin peroxidase method (using ABC kit, Vectastain). In brief, sections were soaked in TBS containing 3% Bovine Serum Albumin (BSA) for 2 h at room temperature to block non-specific tissue antigen and then incubated overnight with the primary antibody in TBS with 1% BSA at 4 °C. Then, sections were washed in TBS + 0.025% Triton X-100, quenching endogenous peroxidase with a 15-min incubation in 0.3% H_2_O_2_ in TBS. Immunohistochemical staining was performed using avidin-biotin-peroxidase with anti-rabbit biotinylated secondary antibody and 3,3′-diaminobenzidine as the chromogen. Slices were mounted on chrome alumgelatinized slides, air-dried for 24 h and then dehydrated with ethanol, cleared in xylene, and coverslipped.

For Ambra1/PV immunofluorescence, slices were permealized with PBS containing 1% Triton X-100 for 10 min at room temperature. Sections were incubated with the primary antibody in PBS containing 2% goat serum for 2 h at 37 °C, transferred for 3 days at 4 °C, washed in PBS, and then incubated for 2 h at room temperature with secondary antibodies. DAPI was used for staining of nuclei.

Primary antibodies: PV for immunohistochemistry (1:5000; Novus, NB120-11427, RRID:AB_791498), PV for immunofluorescence (1:500; Swant, PV235, RRID:AB_10000343), Ambra1 (1:20; Novus Biologicals, NBP1-07124, RRID:AB_1625698). Secondary antibodies: Alexa Fluor™ 488 donkey anti-rabbit IgG (1:300; Molecular Probes), Alexa Fluor™ 555 donkey anti-mouse IgG (1:300; Molecular Probes). The sections were mounted using an anti-fade medium (Fluoromount, Sigma-Aldrich) and examined under a confocal laser-scanning microscope (LSM700, Zeiss). The specificity of the immunohistochemical labeling was confirmed by the omission of primary antibodies and the use of normal serum instead (negative controls).

All images were exported in TIFF format, contrast and brightness were adjusted and final plates were composed with Adobe Illustrator CS3.

### Neuron Counting

Sections processed for immunohistochemistry were used for unbiased estimates of dorsal hippocampus PV interneuron total numbers using the Stereo Investigator System (MicroBrightField Europe e.K.), as described in [36]. For unbiased estimates of hippocampal PV interneuron total numbers, we applied an optical fractionator stereological design (monolateral count) using the Stereo Investigator System (MicroBrightField Europe e.K.). A stack of MAC 5000 controller modules (Ludl Electronic Products, Ltd) was interfaced with an Olympus BX50 microscope with a motorized stage and a HV-C20 Hitachi digital camera with a Pentium II PC workstation. A 3D optical fractionator counting probe (x, y, z dimension of 50 × 50 × 25 μm) was applied. The hippocampus was outlined using a × 5 objective and cells were marked with a × 40 objective. Data collection was done by a researcher blind to the genotype and gender of each animal. PV interneuron numbers were estimated according to the formula:

$$ N=\mathrm{SQ}\ast \frac{1}{\mathrm{ssf}}\ast \frac{1}{\mathrm{asf}}\ast \frac{1}{\mathrm{tsf}} $$

where SQ represents the number of neurons counted in all optically sampled fields of the hippocampus, ssf is the section sampling fraction, asf is the area sampling fraction, and tsf is the thickness sampling fraction.

### Morphological Analysis

Analysis was performed on Nissl-counterstained sections. Slices were mounted on slides, air-dried for 24 h, dehydrated with ethanol, cleared in xylene, and coverslipped.

### TUNEL Labeling

For in situ end labeling of DNA fragmentation (TUNEL) staining, embryos were fixed in Bouin’s solution (5% acetic acid, 9% formaldehyde, 0.9% picric acid), washed in 70% ethanol, dehydrated through successive steps in alcohol at rising concentrations (50, 70, 95, 100%) then cleared in xylene, followed by paraffin embedding. Embryos were cut with a microtome in 5 μm coronal slices. After removal of paraffin with xylene and rehydration in ethanol solutions of decreasing concentration, sections were digested with proteinase K (20 μg/ml, 15 min), washed in distilled water, and exposed briefly to 3% H_2_O_2_. The TUNEL reaction was performed using the Apoptag Plus Peroxidase In-Situ Apoptosis Detection Kit (Millipore). By diaminobenzidine and H_2_O_2_, according to the supplier’s instructions, TUNEL-positive cells were revealed. Then, slices were lightly Nissl-counterstained with cresyl violet.

Cells were defined as apoptotic if they were TUNEL-positive, if they showed nuclei with condensed chromatin or nuclear fragmentation or both. TUNEL-positive cells within MGE were counted with a Leitz DMRB microscope (× 100 magnification).

### Brain Slice Preparation

Parasagittal brain slices (250–300 μm) containing the dorsal hippocampus were obtained as previously described [37]. Briefly, following halothane anesthesia, the brain was removed and sliced with a Leica VT1200S vibratome in chilled bubbled (95% O_2_, 5% CO_2_) artificial cerebrospinal fluid (aCSF; containing in mM: 124 NaCl, 3 KCl, 1.25 NaH_2_PO_4_, 26 NaHCO_3_, 1 MgSO_4_, 2 CaCl_2_, 10 glucose; ~ 300 mOsm, pH 7.4) for field recordings, or with sucrose-based solution for patch-clamp (containing in mM: 3 KCl, 1.25 NaH_2_PO_4_, 26 NaHCO_3_, 10 MgSO_4_, 0.5 CaCl_2_, 25 glucose, 185 sucrose; ~ 300 mOsm, pH 7.4). Slices were incubated in aCSF (1 h, 30–32 °C) and transferred at room temperature for at least 30 min prior to recordings.

### Gamma-Oscillation Recordings

Local field oscillations in the gamma range were obtained from parasagittal hippocampal slices recorded using a multi-electrode array device (MED64; Alpha MED Sciences, Kadoma, Japan). Individual slices were placed over an 8 × 8 array of planar microelectrodes, each 50 × 50 μm in size, with an interpolar distance of 150 μm. Slices were positioned over the multi-electrode array under visual control through an upright microscope (Leica DM-LFS, Leica Microsystems, Wetzlar, Germany), so that most of the planar electrodes were covered by the CA3-CA1 fields. Voltage signals were acquired using the Mobius software (Alpha MED Sciences, Kadoma, Japan), digitized at 20 kHz and low-cut filtered at 1.0 Hz.

The slices were kept submerged in aCSF with a nylon mesh glued to a platinum ring, under a continuous flow of oxygenated aCSF (6 ml/min) at 30 °C, streaming above the hippocampal area. The power spectral density was calculated in the voltage signal acquired from one of the recording electrodes, where higher amplitude oscillations were generated during CCh (20 μM). The signal was down-sampled to 200 Hz and power spectra were calculated using the NeuroExplorer software (Nex Technologies, Littleton, USA) before and during CCh perfusion. We then calculated the integral of the power spectra within the range of the gamma frequencies (25–45 Hz) and the specific peak frequency, at the time when gamma oscillations reached their highest amplitude. Since there was a large variability in the absolute power of oscillations, the power spectral density areas were normalized to that obtained within the same range of frequencies, during the 2 min preceding CCh perfusion.

### Excitatory Postsynaptic Potential Field Recordings

A single brain slice was transferred to a recording chamber of an upright microscope (Axioskop 2-FS; Zeiss, Germany) and completely submerged in aCSF (3–4 ml/min; 30 °C).

Standard field responses were induced by a concentric bipolar stimulating electrode (FHC Inc.; Bowdoin, ME) placed in the Schaffer collateral pathway in the striatum radiatum and afferent stimulation (100 μs duration) was delivered every 30 s.

For eliciting population spikes, a borosilicate glass recording electrode filled with aCSF was positioned in the CA1 stratum pyramidale, 200–300 μm from the stimulating electrode. Input-output curves were obtained by measuring the amplitude of the principal population spike at increasing 10 μA steps of afferent stimulation and all experiments were performed at the intensity yielding a half-maximal response. Following the recording of stable control responses for at least 3 min, 5 and 10 μM (−)-Bicuculline methochloride (Abcam) was bath-applied for 10–12 min to elicit epileptiform bursting consisting of a principal and secondary populations spikes. At least five sweeps were recorded for control conditions and for each bicuculline concentration and the average responses were used for calculating the number of population spikes (with amplitude threshold set at 0.2 mV) and the total duration of the evoked response (measured from the onset of the primary population spike to the offset of the last secondary population spike).

To elicit fEPSPs, an aCSF-filled borosilicate glass recording electrode was positioned in the stratum radiatum of the CA1 hippocampal region, 200–300 μm from the stimulating electrode. Input-output curves were obtained by measuring the fEPSP initial slope at increasing 10 μA steps of afferent stimulation. For LTP experiments, after at least 20 min of test stimulation (at half-maximal intensity, every 30 s) to assess fEPSP slope stability, the slice was challenged with two consecutive trains (1 s) of stimuli at 100 Hz separated by 20 s, followed by test stimulation for at least 1 h. The degree of LTP was evaluated by the fEPSP mean slope at 55–60 min from the conditioning train, normalized to the mean slope during baseline. The field responses were recorded with a MultiClamp 700B amplifier and digitized with Digidata 1322A. Data were sampled at 20 kHz. Traces were obtained by pClamp 9.2 and analyzed using Clampfit 9.2 (all from Molecular Devices; Sunnyvale, CA).

### Patch-Clamp Recordings

Whole-cell patch-clamp recordings from single brain slices were made from the soma of visually identified hippocampal pyramidal neurons located in the CA1 stratum pyramidale. All recordings were performed with a MultiClamp 700B amplifier, using a 3–4 kHz low-pass filter, digitized with Digidata 1322A and computer-saved at a sampling rate of five times the filter frequency. Recording electrodes (3–6 MΩ) were pulled from thin-wall borosilicate glass tubes (TW150F4; World Precision Instruments, Germany). Access resistance was monitored online throughout each experiment and recordings were discarded if either access resistance or holding current increased by more than 25% during the experiment. No liquid junction potential correction was implemented.

For current-clamp recordings, the electrodes were filled with (in mM): 135 K-gluconate, 10 KCl, 2 MgCl_2_, 0.05 CaCl_2_, 0.1 EGTA, 10 HEPES, 4 Mg-ATP, 0.3 Na-GTP (~ 275–285 mOsm, pH 7.2). The voltage threshold for action potential (AP) discharge was evaluated with short-duration (50 ms) depolarizing current steps of increasing amplitudes (5 pA increments). The threshold was determined by the maximum of the second derivative of membrane potential by time, corresponding to the inflection point at the start of the AP. AP amplitude was defined as the difference between resting membrane potential (*V*_rest_) and peak voltage; the full-width at half-maximum amplitude (FWHM) was the time difference at half-maximal AP amplitude. Rise and decay slope of AP was the 10–90% rate of membrane potential change during the rise and decay phase of the AP, respectively. Longer steps (600 ms, 50 pA increments) were used to obtain current/voltage (I/V) curves at sub-threshold responses and AP number calculations at supra-threshold responses. Input resistance (*R*_in_) was calculated from the slope after linear regression of I/V curves at steady-state sub-threshold responses. Sag ratio was the ratio of the steady-state versus peak potential during sub-threshold responses to − 200 pA current injections. All current-clamp recordings were obtained from a resting membrane potential kept to − 65 mV by current injection.

eEPSCs were evoked by stimulating in the stratum radiatum and currents were recorded at − 70 mV holding potential using a Cs-based filling solution containing (in mM): 117.5 Cs-methanesulfonate, 15.5 CsCl, 8 NaCl, 1.25 NaH_2_PO_4_, 10 HEPES, 0.25 EGTA, 10 TEA-Cl, 2.5 QX314-Cl, 4 Mg-ATP, 0.3 Na-GTP (~ 290 mOsm, pH 7.2), in the presence of bath-perfused picrotoxin (50 μM; Tocris) and CGP55845 (1 μM; Abcam). To calculate the AMPA/NMDA ratio, single eEPSCs were evoked at half-maximal intensity, the AMPAR component was assessed by the peak of the inward current measured at − 70 mV holding potential, while the NMDAR component was estimated by the amplitude of the outward current of the same synaptic response at +40 mV holding potential, 70 ms after the stimulation artifact.

eIPSCs (− 70 mV holding potential) were recorded using pipettes filled with (in mM): 140 CsCl, 1 MgCl_2_, 10 HEPES, 2.5 QX314-Cl, 4 Mg-ATP (~ 290 mOsm, pH 7.2), in the presence of NBQX (10 μM; Tocris), D-AP5 (50 μM; Tocris), and CGP55845 (1 μM; Abcam). Inhibitory currents were evoked by placing the stimulating electrode in close proximity to the recorded neuron, either in the stratum radiatum to evaluate dendritic inhibition, or in the stratum pyramidale to evaluate perisomatic inhibition. Perisomatic eIPSCs (− 70 mV) from perisomatic-targeting interneurons were recorded in the presence of 1 μM WIN55,212-2((*R*)-(+)-[2,3-dihydro-5-methyl-3-(4-morpholinylmethyl)pyrrolo[1,2,3-*de*]-1,4benzoxazin-6-yl]-1-naphthalenylmethanone mesylate; Tocris) to block GABA release from cholecystokinin (CCK) basket cells.

For all the recordings, the stimulating electrode was a monopolar glass electrode filled with aCSF and stimuli (100 μs duration every 30 s) were applied with an ISO-Flex stimulus isolator (A.M.P.I, Israel). For the input/output curves of eEPSCs and eIPSCs, the stimulus intensity referred to as 1× was determined as the intensity that elicited a response between 15 and 20 pA. Stimulation was increased in a multiplicative manner to develop the input/output curves. Overall, stimulus intensity ranged from 20 to 110 μA. Paired-pulse ratios (PPR) were calculated using pairs of afferent stimuli at half-maximal intensity at different paired-pulse intervals and are expressed as peak of the second/first current.

AMPAR-mediated whole-cell currents (− 70 mV) were recorded in the presence of bath-applied AMPA (1 μM; Abcam) and cyclothiazide, to prevent fast receptor desensitization (20 μM; Abcam), together with D-AP5 (50 μM; Abcam) and lidocaine (500 μM; Abcam). NMDAR-mediated currents (+ 40 mV) were recorded in NMDA (10 μM; Abcam), glycine (10 μM; Abcam), NBQX (10 μM; Abcam), and lidocaine (500 μM; Abcam). The experiments were performed in the presence of picrotoxin (100 μM; Tocris).

Miniature excitatory (mEPSCs) and inhibitory (mIPSCs) postsynaptic currents from hippocampal pyramidal neurons were recorded in voltage-clamp mode (at − 70 mV) using the same solutions and drugs as for evoked currents, in the presence of bath-applied lidocaine (500 μM; Abcam). Single events were detected with Mini Analysis (Synaptosoft Inc., USA) and analyzed for amplitude and inter-event interval (IEI). For the recording segments chosen for analysis, both parameters were tested for time stability using Spearman’s rank order correlation test [38] and segments of events that showed time instability during the experiment were excluded from further analysis. The median values of IEI and amplitude of at least 400 events were analyzed for each experiment.

### Spine Morphology

For Golgi-Cox staining, mice were perfused transcardially with 0.9% saline and samples were impregnated in a Golgi-Cox solution (1% potassium dichromate, 1% mercuric chloride and 0.8% potassium chromate) at room temperature for 6 days. On the 7th day, brains were transferred in 30% sucrose solution and sectioned with a vibratome. Coronal sections (100 μm) were stained through consecutive steps in water (1 min), ammonium hydroxide (30 min), water (1 min), developer solution (Kodak fix 100%, 30 min), and water (1 min). Sections were then dehydrated through successive steps in alcohol at rising concentrations (50, 75, 95, and 100%) and covered with slide coverslips. Spine analysis was performed on dendritic segments of basal and apical dendrites of CA1 pyramidal neurons. Neurons were first identified with a light microscope (Leica DMLB) under a ×20 objective. Subsequently, quantification of dendritic spines was done online under ×100 magnification using a Qimaging Qicam Fast1394 camera connected to the microscope. From each brain, five neurons were selected and on each neuron, five 30–100 μm dendritic segments were randomly selected for spine counts. Only protrusions with a clear connection of the head of the spine to the shaft of the dendrite were counted as spines using the Neurolucida software (v11, MBF Bioscience). Analysis of dendritic spine size was carried out after random selection of spines, by measuring spine head diameter parallel to the dendrite using the ImageJ software. Spine head width values were expressed as cumulative frequencies.

### Statistical Analysis

Analysis was performed using Prism (GraphPad Software v5). Behavioral data for social interaction were analyzed with two-way ANOVAs with genotype and vocalization numbers or sniffing duration as independent factors, followed by Tukey’s post-hoc test. Behavioral data from the three-chamber task were analyzed with one-way ANOVAs (Str versus Obj and Fam versus non-Fam). Cumulative frequencies of spine head diameters were compared with the Kolmogorov-Smirnov test. Ambra1 protein levels were compared with two-way ANOVAs for genotype and gender, with Bonferroni’s post-hoc test. All other data were analyzed using two-tailed unpaired *t* test (WT versus Ambra1^+/−^). *P* < 0.05 indicates statistical significance (shown in Figures as *P < 0.05, **P < 0.01, ***P < 0.001). Details on statistical analysis can be found in figure legends.

### Sample Size

The target number of samples in each group for immunohistochemical, immunofluoscent, stereological, electrophysiological, biochemical, behavioral, and morphological experiments was determined on the basis of numbers reported in published studies.

### Randomization and Blinding

All randomization was performed by an experimenter. Following separation of all available animals into four groups based on genotype and sex (WT females, WT males, Ambra1^+/−^ females, Ambra1^+/−^ males), a random number was assigned for each animal; randomization for the animals used in experiments was achieved using a random number table. A similar method was used for unfamiliar C57BL/6J female mice used in the behavioral tests.

All data were collected by researchers blind to the genotype and sex of each animal.

#### Data Availability

The data that support the findings of this study are available from the corresponding author (Marcello D’Amelio; m.damelio@unicampus.it).

## Results

### Ambra1^+/−^ Female Mice Display Impaired Social Interaction

**Fig. 1:**
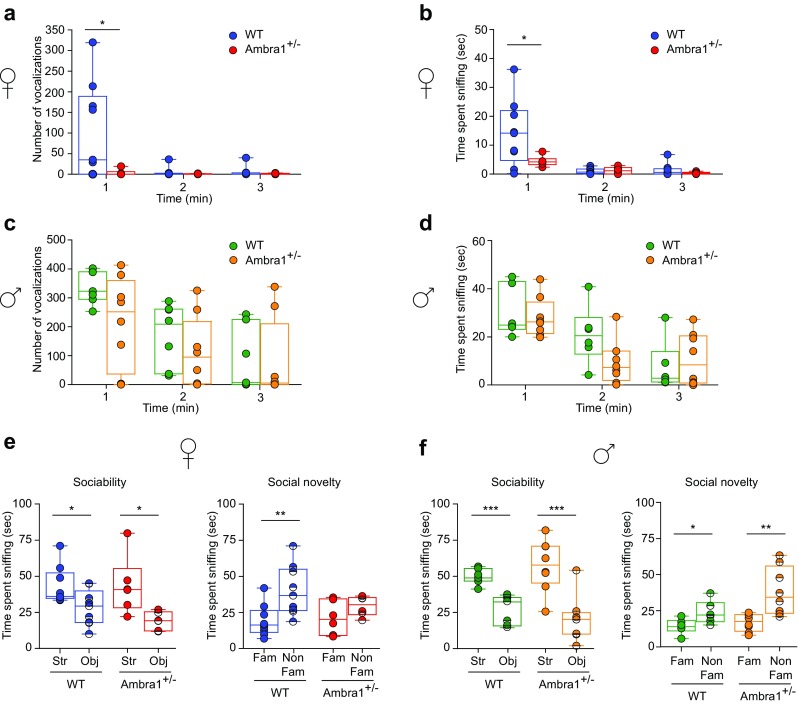
Female Ambra1^+/−^ mice show an autism-like deficiency in social interaction **a**, **b** Number of ultrasonic vocalizations (**a**) and total time spent sniffing (**b**) for female WT and Ambra1^+/−^ mice during a 3-min interaction. Female Ambra1^+/−^ mice exhibit fewer ultrasonic vocalizations and reduced sniffing duration during the 1st minute of interaction (*n* = 9 WT, 6 Ambra1^+/−^; **a**: two-way ANOVA for vocalizations×genotype for the 3 min duration: *F*_2,26_ = 4.395, *P* = 0.023; *P < 0.050 with Tukey’s post hoc test for the 1st minute; **b**: two-way ANOVA for sniffing×genotype: *F*_2,26_ = 4.579, *P* = 0.020; *P < 0.050 with Tukey’s for the 1st minute). **c**, **d** Number of ultrasonic vocalizations (**c**) and time spent sniffing (**d**) during social interaction of males with sexually receptive C57BL/6J females. No differences are observed between genotypes (*n* = 6 WT, 8 Ambra1^+/−^; **c**: two-way ANOVA for vocalizations×genotype: *F*_2,24_ = 1.923, *P* = 0.168; **d**: two-way ANOVA for sniffing×genotype: *F*_2,24_ = 0.072, *P* = 0.795). **e** Sociability and social novelty phase for females. Left: All females spent more time sniffing the stranger mouse (Str) compared to the object (Obj) during sociability phase (*n* = 9 WT, 6 Ambra1^+/−^ females; one-way ANOVA: WT, *F*_1,16_ = 6.352, *P = 0.023; Ambra1^+/−^, *F*_1,10_ = 8.218, *P = 0.017). Right: Ambra1^+/−^ females fail to show preference for the non-familiar (Non-Fam) over the familiar (Fam) mouse during social novelty phase (*n* = 9 WT, 6 Ambra1^+/−^; one-way ANOVA: WT, *F*_1,16_ = 9.431, **P = 0.007; Ambra1^+/−^, *F*_1,10_ = 2.130, *P* = 0.175). **f** Sociability and social novelty phase in males. Regardless of genotype, all males spent more time sniffing the Str mouse (*n* = 6 WT, 8 Ambra1^+/−^; one-way ANOVA: WT, *F*_1,10_ = 27.441, ***P = 4.00 × 10^−4^; Ambra1^+/−^, *F*_1,14_ = 19.316, ***P = 6.00 × 10^−4^) and showed preference for the Non-Fam animal (*n* = 6 WT, 8 Ambra1^+/−^; one-way ANOVA: WT, *F*_1,10_ = 6.249, *P* = *0.032; Ambra1^+/−^, *F*_1,14_ = 11.296, **P = 0.004). In all figures, in box-and-whisker plots, the centre lines denote medians, edges are upper and lower quartiles, whiskers show minimum and maximum values, and points are individual experiments

Behavioral deficits in rodents are often reflected by a reduction in the exploratory behavior toward social stimuli [39]. To assess whether 2–3-month-old Ambra1^+/−^ mice display social deficits, we performed female-female and male-female social interaction tests and analyzed the ultrasonic emissions and sniffing responses of the test animals. During the female-female social interaction test, Ambra1^+/−^ females emitted fewer ultrasonic vocalizations (Fig. 1a) compared to WT mice and spent less time interacting with the unfamiliar female during the first minute of the social interaction (Fig. 1b). On the other hand, during the male-female social interaction test, the number of vocalizations emitted by males (Fig. 1c) and sniffing duration (Fig. 1d) did not differ from WT.

To quantify sociability and preference for social novelty, we used a three-chamber task [33]. During sociability phase, female and male mice of both genotypes showed stronger preference for the stranger mouse compared to the object (left panels, Fig. 1e,f). However, during the social novelty phase, although WT mice preferred socializing more with the unfamiliar mouse, Ambra1^+/−^ female mice failed to show such preference and spent equal amount of time sniffing both animals (right, Fig. 1e). By contrast, male mice of both genotypes showed preference for the unfamiliar mouse (right, Fig. 1f). These results suggest that *Ambra1* haploinsufficiency impairs sociability and social communication only in females.

### Reduction of Hippocampal PV Interneurons in Ambra1^+/−^ Females

**Fig. 2:**
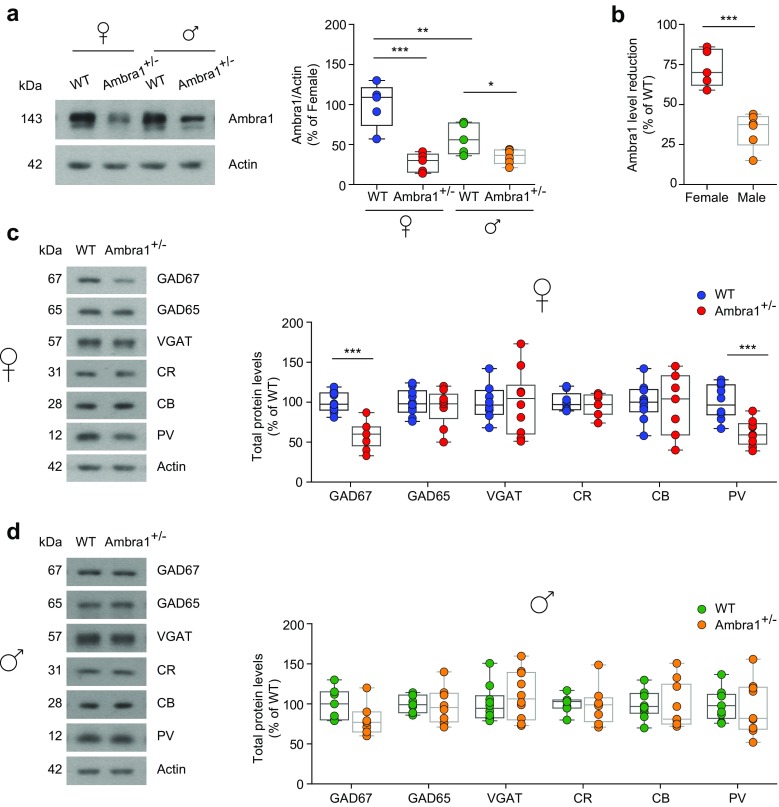
Female Ambra1^+/−^ mice have stronger reduction in hippocampal Ambra1 protein levels and show reductions in GABAergic neuron markers. **a** Representative immunoblots of total Ambra1 protein extracted from hippocampi of female and male Ambra1^+/−^ and age-matched WT mice, probed with the Ambra1 antibody. Actin was used as loading control. The plot shows densitometric quantification of changes in gray values, expressed as % of female WT (females: *n* = 5 WT and 5 Ambra1^+/−^; males: *n* = 5 WT and 6 Ambra1^+/−^; two-way ANOVA for gender×genotype: *F*_1,17_ = 9.994, *P* = 0.006; Bonferroni’s post hoc test: WT female-Ambra1^+/−^ female, ***P < 1.00 × 10^−4^; WT female-WT male, **P < 0.01; WT male- Ambra1^+/−^ male, *P < 0.05). **b** Relative reduction (from the respective WT of each gender) of hippocampal Ambra1 protein in female and male Ambra1^+/−^ mice (*n* as in **a**; two-tailed unpaired *t* test between genders: ***P = 3.00 × 10^−4^). **c, d** Representative immunoblots of total GABAergic neuron proteins extracted from hippocampi of female (**c**) and male (**d**) mice, probed with the indicated antibodies and densitometric quantification of changes in gray values (*n* = 9 mice per genotype and per gender). Ambra1^+/−^ female mice show reduced levels of GAD67 (two-tailed unpaired *t* test: ***P < 1.00 × 10^−4^) and PV (***P = 4.00 × 10^−4^)

To understand the neuropathology underlying the altered phenotype of Ambra1^+/−^ female mice, we focused on the hippocampus. To study the extent of the *Ambra1* haploinsufficiency in Ambra1^+/−^ mice, we measured the hippocampal Ambra1 protein levels in 2–3-month-old animals. Protein levels were significantly reduced in Ambra1^+/−^ mice of both genders compared to WT littermates (Fig. 2a). Interestingly, Ambra1 protein expression in WT females was significantly higher compared to males and the relative protein reduction in Ambra1^+/−^ mice was more pronounced in females (Fig. 2b).

Recent data support a strong link between changes in the number and/or function of interneurons and behavioral alterations in neurodevelopmental diseases, including schizophrenia and ASDs [13, 14, 40, 41]. Considering the altered phenotype in Ambra1^+/−^ females, we analyzed the hippocampal levels of different GABAergic neuron markers, including glutamic acid decarboxylase (GAD) enzymes, vesicular GABA transporter (VGAT) and the proteins PV, calbindin (CB), and calretinin (CR), used for classifying interneurons into three subsets based on calcium-binding protein expression [42, 43]. Interestingly, we found that Ambra1^+/−^ females showed significant reductions in the levels of GAD67 and PV (Fig. 2c) whereas Ambra1^+/−^ males showed protein levels similar to WT (Fig. 2d).

**Fig. 3:**
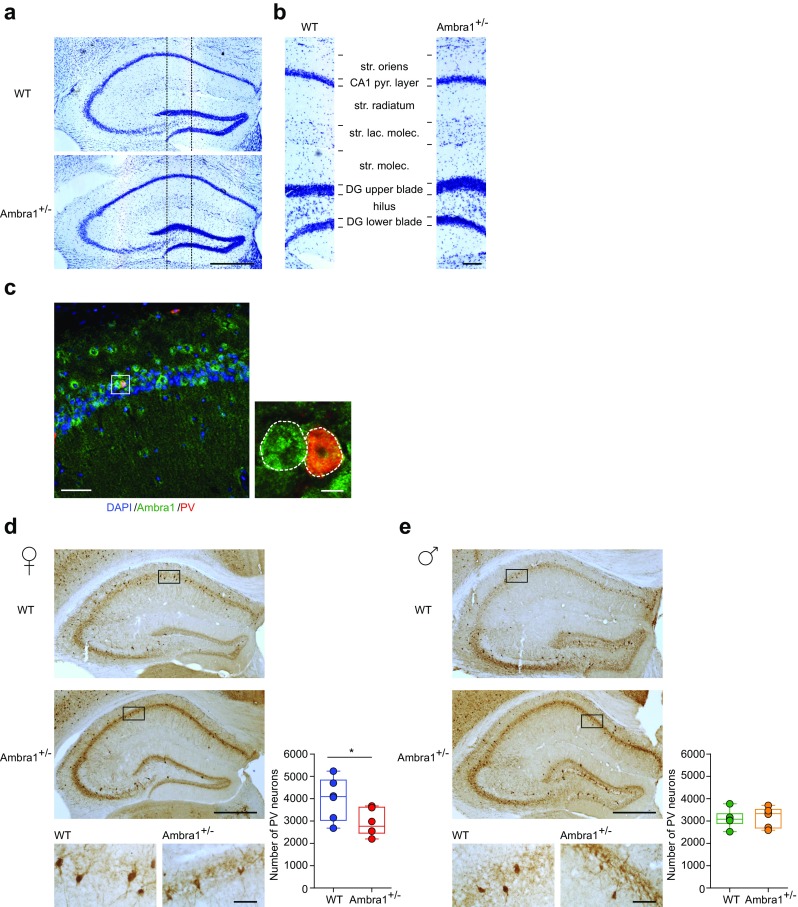
Female Ambra1^+/−^ mice show reduced numbers of hippocampal PV interneurons. **a** Nissl staining of dorsal hippocampus coronal sections from WT and Ambra1^+/−^ female mice (scale bar: 500 μm). **b** Subdivision of hippocampal layers (stratum oriens, CA1 pyramidal layer, stratum radiatum, stratum lacunosum moleculare, stratum moleculare, dentate gyrus upper blade, hilus, dentate gyrus lower blade), corresponding to the area depicted in **a** (scale bar: 100 μm). Ambra1^+/−^ females show normal hippocampal layering (*n* = 3 mice per genotype). **c** Double labeling for Ambra1 (green) and PV (red) in the CA1 from a WT female; sections were DAPI-counterstained (blue) for neuronal nuclei (scale bar: 50 μm). The higher magnification image (scale bar, 5 μm) from the stratum pyramidale shows a PV neuron (right; merge in orange) and an unidentified neuron (left), both expressing Ambra1. The DAPI signal is omitted for clarity and neuron borders are indicated by a dash line (*n* = 3 mice; three sections per animal). **d**, **e** Immunohistochemical labeling of PV in a coronal brain section of dorsal hippocampus obtained from female (**d**) and male (**e**) mice (scale bars: 500 μm). On the right are higher magnification images (scale bars: 50 μm). The plots show stereological quantification of PV interneurons (*n* = 6 mice per genotype and per gender; 14 sections per animal). The reduction in PV interneuron numbers is selective for female Ambra1^+/−^ (two-tailed unpaired *t* test: *P = 0.043)

To investigate whether reductions in these GABAergic markers in Ambra1^+/−^ females were linked to abnormalities in the hippocampal anatomical structure, we performed a morphological analysis on sections from female mice, stained for the pan-neuronal marker Nissl. We observed a similar anatomical hippocampal structure in both genotypes, excluding apparent structural defects in the Ambra1^+/−^ female hippocampus (Fig. 3a,b).

The evidence that Ambra1 is expressed in hippocampal PV interneurons (Fig. 3c) prompted us to investigate, using an anti-PV antibody, whether the selective reduction of GAD67 and PV in Ambra1^+/−^ females might reflect a reduction in PV interneuron numbers. Indeed, we observed a reduced number of hippocampal PV interneurons in Ambra1^+/−^ female mice (Fig. 3d) and, in line with data on protein markers, this deficit was absent from Ambra1^+/−^ males (Fig. 3e).

### Reduced Power of CA3-CA1 Gamma-Oscillations in Female Ambra1^+/−^ Mice

**Fig. 4:**
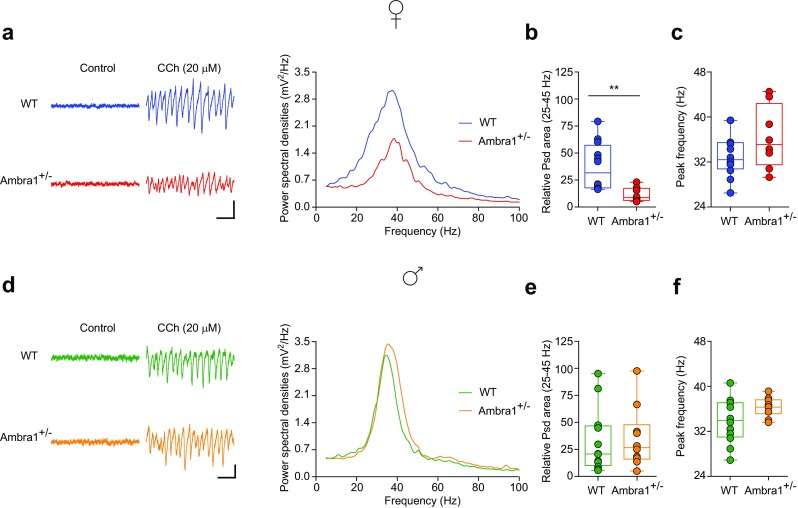
Reduction in the power of hippocampal gamma-oscillations in female Ambra1^+/−^ mice. **a** Traces are local field potentials recorded with a multi-electrode array device in the hippocampal pyramidal layer of a female WT and an Ambra1^+/−^ mouse before and during CCh bath perfusion (scale bars: 100 ms, 30 μV). On the right are power spectra for these signals during CCh, showing a reduced power in the Ambra1^+/−^ mouse. **b**, **c** The box-and-whisker plots indicate the change in power spectral density (Psd) area (**b**) in the gamma-frequency range (25–45 Hz; two-tailed unpaired *t* test: **P = 0.004) and peak frequency (**c**) in 20 μM CCh (*n* = 12 WT slices and 8 Ambra1^+/−^ slices). **d–f** Same as in **a–c**, showing unchanged gamma-oscillations in male Ambra1^+/−^ compared to WT mice (*n* = 12 WT and 10 Ambra1^+/−^ slices)

Fast-spiking PV interneurons are essential for generating cortical and hippocampal gamma-oscillations [44–48], and abnormalities in PV interneurons have been associated with dysfunctions in gamma-frequency synchronization in autism and schizophrenia [49, 50]. Due to the reduced PV interneuron numbers in Ambra1^+/−^ females, we investigated whether hippocampal gamma-oscillations were also altered, using carbachol (CCh) to induce gamma-oscillations in vitro [51, 52]. In line with the selective PV interneuron reduction in Ambra1^+/−^ female mice, we observed a reduction in the power of CCh-induced gamma-oscillations in the frequency range of 25–45 Hz in female (Fig. 4a,b) but not male Ambra1^+/−^ mice (Fig. 4d, e), whereas the peak frequency of oscillations was unchanged in both genders (Fig. 4c, f). Thus, the hippocampal oscillatory circuit is impaired in Ambra1^+/−^ female mice, although it remains intact in males.

### Reduced Inhibition/Excitation Ratio in Ambra1^+/−^ Female Mice

**Fig. 5:**
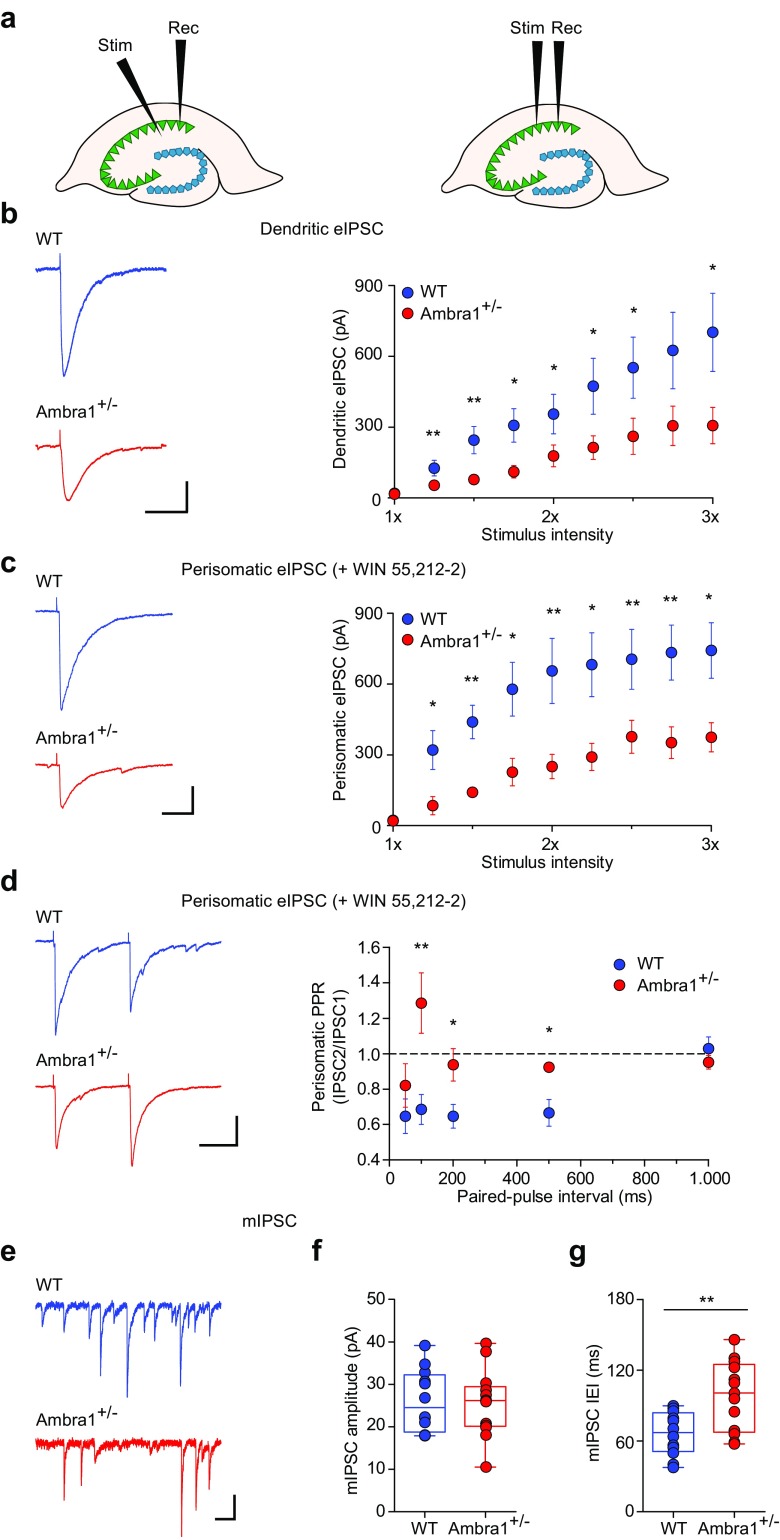
Female Ambra1^+/−^ mice show reduced inhibitory synaptic transmission onto CA1 pyramidal neurons. **a** Schematic of a dorsal hippocampus slice showing the positioning of the stimulating and recording electrodes during recordings of dendritic (left) and perisomatic eIPSCs (right). **b** Examples of dendritic eIPSCs at half-maximal Schaffer collateral stimulation intensity from a female WT and Ambra1^+/−^ CA1 pyramidal neuron (− 70 mV; scale bars: 100 ms, 100 pA), and mean input/output curve (± sem) for eIPSCs (*n* = 17 WT and 16 Ambra1^+/−^ cells) in response to increasing afferent stimulation. eIPSCs are reduced in Ambra1^+/−^ females (two-tailed unpaired *t* test for the indicated intensities: *P < 0.050, **P < 0.010). **c** Traces of perisomatic eIPSCs from CA1 pyramidal neurons (− 70 mV; scale bars: 20 ms, 100 pA) recorded at half-maximal stimulation in the stratum pyramidale in the presence of WIN55,212-2 (1 μM), and mean input/output curve (± sem; *n* = 8 WT and 7 Ambra1^+/−^ cells) showing that perisomatic eIPSCs are reduced in Ambra1^+/−^ females (two-tailed unpaired *t* test for the indicated intensities: *P < 0.050, **P < 0.010). **d** Example paired-pulse responses (100 ms interval) of perisomatic eIPSCs from a female WT and Ambra1^+/−^ pyramidal neuron (− 70 mV; scale bars: 50 ms, 100 pA) at half-maximal stimulation in the stratum pyramidale in the presence of WIN55,212-2. The PPR (± sem) is increased in Ambra1^+/−^ females (two-tailed unpaired *t* test for the indicated intervals: *P < 0.050, **P < 0.010; *n* = 8 WT and 7 Ambra1^+/−^ cells). **e** Representative mIPSCs from female WT and Ambra1^+/−^ pyramidal neurons (− 70 mV; scale bars: 100 ms, 20 pA). **f**, **g** The mean mIPSC amplitude is unchanged in female Ambra1^+/−^ mice (**f**), but the IEI (**g**) is reduced (*n* = 12 WT and 13 Ambra1^+/−^ cells; two-tailed unpaired *t* test: **P = 0.003)

Different types of PV interneurons innervate distinct sub-cellular domains of pyramidal neurons, thus mediating both dendritic and perisomatic inhibition [53, 54]_._ To test the effects of reduction of female Ambra1^+/−^ PV interneurons on the inhibition/excitation ratio in the hippocampus, we recorded eIPSCs from CA1 pyramidal neurons following stimulation in the stratum radiatum (to evaluate dendritic-targeting inhibition), or in the stratum pyramidale (to evaluate perisomatic inhibition; Fig. 5a).

First, we measured dendritic eIPSC input/output curves by plotting the evoked current in response to a range of Schaffer collateral stimulation intensities. We observed a significant reduction in the amplitude of eIPSCs in Ambra1^+/−^ females across the whole range of stimulation (Fig. 5b), indicating a decrease in the magnitude of dendritic inhibition. We did not observe differences in the input resistance (Suppl. Fig. 1a) or excitability (Suppl. Fig. 1b, c) of CA1 pyramidal neurons between the two genotypes (Suppl. Table 1).

To confirm that the reduction of the inhibitory drive on principal neurons is due to reduced PV interneuron numbers in Ambra1^+/−^ mice, we next recorded perisomatic eIPSCs from pyramidal neurons by stimulating in the stratum pyramidale. These perisomatic eIPSCs are primarily from PV-expressing perisomatic-targeting interneurons with axons in the stratum pyramidale, including fast-spiking basket and axo-axonic cells, as well as from CCK-expressing regular-spiking basket cells [55, 56]. We isolated PV neuron-derived perisomatic eIPSCs by pharmacologically blocking GABA release from CCK neurons with WIN55,212-2, an agonist of cannabinoid CB1 receptors that are expressed in CCK but not in PV interneurons [57]. We observed an approximately twofold lower perisomatic eIPSC amplitude in Ambra1^+/−^ females (Fig. 5c). Additionally, the paired-pulse ratio (PPR) of perisomatic eIPSC in the presence of WIN55,212-2, was significantly increased at intervals of 100–500 ms, suggesting a lower GABA release probability in Ambra1^+/−^ mice (Fig. 5d). To confirm that the reduced inhibition in Ambra1^+/−^ females is due to a presynaptic and not a postsynaptic mechanism, we recorded miniature inhibitory postsynaptic currents (mIPSCs) from CA1 principal cells (Fig. 5e). The mIPSC amplitude did not differ between WT and Ambra1^+/−^ mice, suggesting no change in the responsiveness of postsynaptic receptors for GABA (Fig. 5f). There was, however, a significant decrease in the mIPSCs frequency (Fig. 5g), in line with the lower GABA release probability and loss of presynaptic interneurons.

**Fig. 6:**
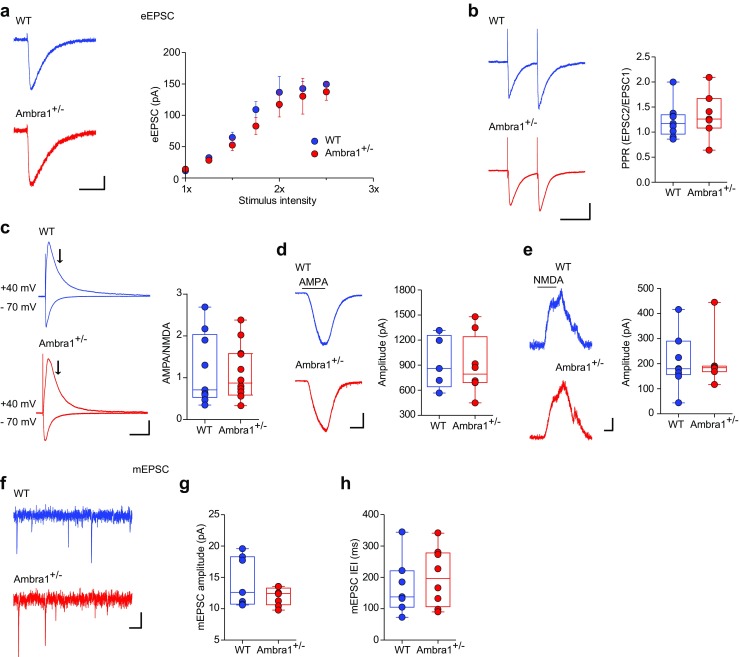
Female Ambra1^+/−^ mice show normal excitatory synaptic transmission onto CA1 pyramidal neurons. **a** Representative traces at half-maximal stimulation intensity and mean input/output curve (± sem) for eEPSCs recorded from female WT and Ambra1^+/−^ CA1 pyramidal neurons held at − 70 mV (*n* = 7 WT and 10 Ambra1^+/−^ cells; scale bars: 50 ms, 20 pA) in response to increasing Schaffer collateral stimulation intensity. **b** The traces are example paired-pulse responses (100 ms interval, − 70 mV; scale bars: 100 ms, 50 pA) of eEPSCs recorded from CA1 pyramidal neurons at half-maximal stimulation. The PPR of excitation is unchanged between the two genotypes (*n* = 9 WT and 7 Ambra1^+/−^ cells). **c** Example traces of AMPAR- and AMPAR+NMDAR-mediated eEPSCs at − 70 and + 40 mV, respectively, from a female WT and an Ambra1^+/−^ CA1 pyramidal neuron (scale bars: 100 ms, 40 pA). The AMPA/NMDA ratio for each experiment was calculated from peak AMPAR versus NMDAR currents at 70 ms (arrows) from the stimulus artifact (*n* = 9 WT and 12 Ambra1^+/−^ cells). **d**, **e** Representative AMPA- (**d**) and NMDA-induced (**e**) whole-cell currents (scale bars: **d**, 2 min, 200 pA; **e**, 1 min, 50 pA) and plots of mean responses, recorded from CA1 pyramidal neurons held at − 70 and +40 mV, respectively. Bars above traces show the duration of bath application. No differences are observed between WT and Ambra1^+/−^ mice (**d**: *n* = 5 WT and 8 Ambra1^+/−^ cells; **e**: *n* = 9 WT and 7 Ambra1^+/−^ cells). **f–h** Representative AMPAR-mediated mEPSCs (− 70 mV; scale bars: 100 ms, 5 pA) from female WT and Ambra1^+/−^ CA1 pyramidal neurons (**f**), with plots showing mean mEPSCs amplitude (**g**) and IEI (**h**; *n* **=** 7 WT and 8 Ambra1^+/−^ neurons)

To investigate whether the excitatory neurotransmission onto CA1 pyramidal neurons is also altered in Ambra1^+/−^ females, we recorded eEPSCs following Schaffer collateral stimulation. The input/output curves showed that eEPSCs from Ambra1^+/−^ females were unchanged compared to WT animals (Fig. 6a). Additionally, the PPR (100 ms interval) at half-maximal intensity suggested similar glutamate release probability (Fig. 6b) and the AMPA/NMDA ratio was unchanged between genotypes (Fig. 6c). This was further confirmed by whole-cell recordings of currents induced by bath application of AMPA (Fig. 6d) or NMDA **(**Fig. 6e). Finally, the amplitude and frequency of miniature excitatory postsynaptic currents (mEPSCs) did not differ between genotypes (Fig. 6f–h). Altogether, our data indicate that Ambra1^+/−^ female mice demonstrate a reduced hippocampal inhibition/excitation ratio due to reduced inhibitory inputs to principal neurons, whereas excitatory inputs from Schaffer collaterals appear to be intact.

### Increased Hyperexcitability and CA3-CA1 Synaptic Plasticity in Ambra1^+/−^ Females

**Fig. 7:**
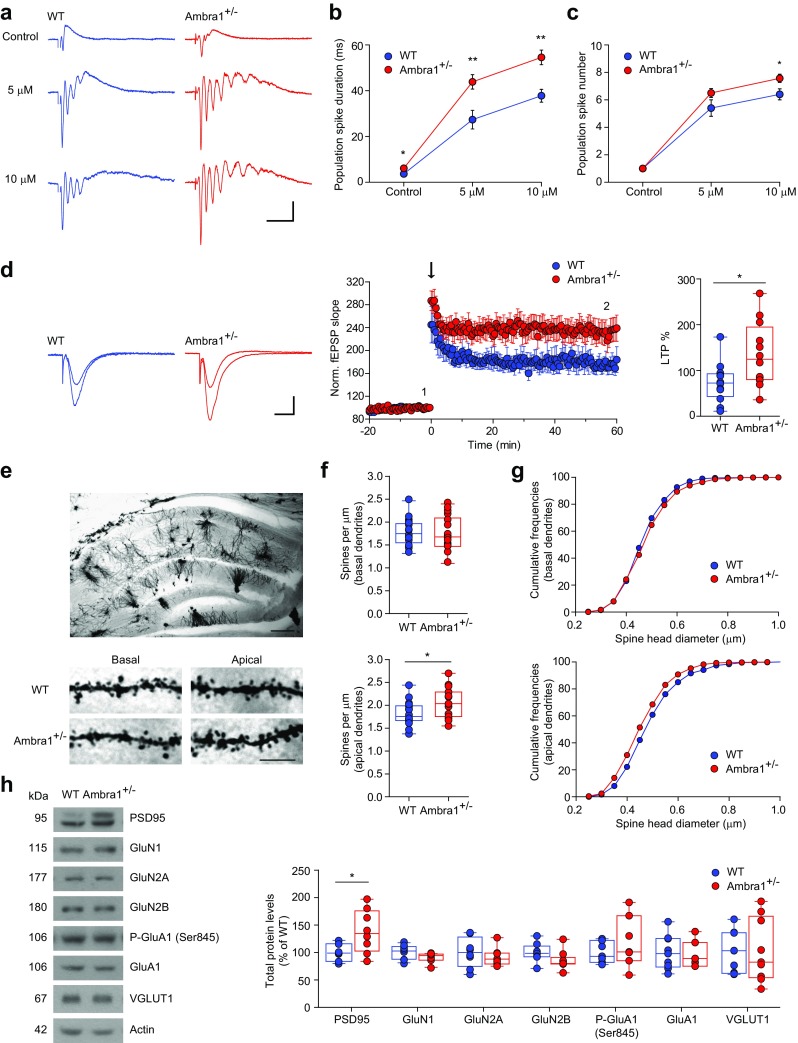
Female Ambra1^+/−^ mice show increased epileptiform activity and synaptic plasticity and increases in apical dendrite spine density. **a** Examples of populations spikes recorded in the stratum pyramidale following Schaffer collateral half-maximal stimulation under control conditions and in the presence of 5 and 10 μM bicuculline in slices from female mice. Stimulus artifacts are truncated for clarity (scale bars: 20 ms, 0.5 mV). Both the duration of the evoked responses (**b**) and the number of population spikes (**c**) is increased in Ambra1^+/−^ (*n* = 5 WT and 8 Ambra1^+/−^ slices; **b**: *P = 0.048 for control, **P = 0.008 for 5 μM, **P = 0.004 for 10 μM; **c**: *P = 0.037 with two-tailed unpaired *t* test). **d** Running plots of normalized CA3-to-CA1 fEPSP mean slope (± sem) recorded in the CA1 stratum radiatum in slices from female mice. The arrow indicates when a high frequency conditioning train was delivered on the Schaffer collaterals. The traces (scale bars: 10 ms, 0.3 mV) are superimposed fEPSPs recorded during baseline (1) and 1 h after the high-frequency train (2). The plot indicates the degree of potentiation, measured as fEPSP slope increase from baseline, 55–60 min after the train (*n* = 12 slices; two-tailed unpaired *t* test: *P = 0.018). See also Suppl. Fig. 2 for input-output curve in females and Suppl. Fig. 3a for LTP recordings in male mice. **e** Top, representative photomicrograph of a Golgi-stained section of the dorsal hippocampus (scale bar: 50 μm). Bottom, segments of basal and apical dendrites of CA1 pyramidal neurons in WT and Ambra1^+/−^ female mice (scale bar: 5 μm). **f** Spine density in females, (*P = 0.023; 18 WT and 17 Ambra1^+/−^ basal dendritic segments, 18 WT and 15 Ambra1^+/−^ apical dendritic segments). **g** Cumulative frequencies of spine head diameter in basal and apical dendrites of pyramidal neurons from female mice (basal, *P* = 0.018, Z = 1.532; apical, *P* = 1.00 × 10^−5^, Z = 2.490; with Kolmogorov-Smirnov test). See also Suppl. Fig. 3b for male mice. **h** Representative immunoblots of total proteins extracted from hippocampi of female mice, probed with the indicated antibodies. The plot shows densitometric quantification of changes in gray values, expressed as % of WT (*n* = 8 mice per genotype and per gender). Ambra1^+/−^ females show increased levels of PSD95 (two-tailed unpaired *t* test: PSD95, *P = 0.032)

To evaluate the consequences of the inhibition/excitation unbalance in Ambra1^+/−^ females, we investigated the predisposition of CA1 neurons to hyperexcitability, in the form of multiple population spikes. Orthodromic Schaffer collateral stimulation always elicited a single population spike in WT slices, whereas in Ambra1^+/−^ slices, the principal spike was occasionally followed by secondary population spikes, resulting in a longer duration of the evoked response (Fig. 7a,b). Bath application of the GABA_A_R antagonist bicuculline resulted in the insurgence of multiple population spikes, typical of an epileptic-like activity, in all recorded slices, and increased the duration of the total response. However, compared to WT slices, the bicuculline-induced effect was significantly stronger in Ambra1^+/−^ slices (Fig. 7a–c), in line with reduced inhibition onto pyramidal neurons.

Next, we investigated long-term potentiation (LTP) of CA3-CA1 synapses by stimulating the Schaffer collaterals and recording field excitatory postsynaptic potentials (fEPSPs) in the CA1 dendritic region. LTP was significantly higher in Ambra1^+/−^ female mice compared to WT (Fig. 7d, Suppl. Fig. 2), in agreement with the reduction of both perisomatic and dendritic inhibition on pyramidal neurons. Correspondingly, Ambra1^+/−^ males that showed unchanged PV interneuron numbers also showed no difference in LTP magnitude compared to WT littermates (Suppl. Fig. 3a).

Previous studies reported structural abnormalities of dendritic spines in patients and animal models of neurodevelopmental disorders, pointing to increased spine numbers, likely due to incomplete pruning or exaggerated spine formation during neurodevelopment [58, 59]. Additionally, impaired autophagy was shown to cause autism-like pruning deficits [60]. We next asked whether changes in dendritic spines might contribute to the enhanced LTP in Ambra1^+/−^ female mice by measuring spine density and head diameter in Golgi-stained CA1 pyramidal neurons (Fig. 7e). The spine density along basal dendrites in the stratum oriens was unaltered in Ambra1^+/−^ mice of both genders compared to WT. However, we detected an increase in the density of apical dendrite spines in the stratum radiatum in Ambra1^+/−^ female (Fig. 7f) but not male mice (Suppl. Fig. 3b). The spine head diameters were different for both basal and apical dendrites between WT and Ambra1^+/−^ female mice (Fig. 7g).

In line with the increased apical dendrite spine density in Ambra1^+/−^ females, analysis of hippocampal total protein extracts revealed increase levels of PSD95, a protein responsible of synaptic structure and plasticity (Fig. 7h). We did not observe changes in the levels of NMDAR or AMPAR subunits in Ambra1^+/−^ mice, in line with electrophysiological observations, or in the levels of the vesicular glutamate transporter 1 (VGLUT1). Our data suggest that *Ambra1* haploinsufficiency in Ambra1^+/−^ female mice might contribute to perturbations in the pruning, formation, or recycling of pyramidal neuron dendritic spines, leading to an overall increase in spine numbers, stronger bicuculline-induced epileptiform activity, and enhanced synaptic plasticity following high-frequency stimulation.

### Interneuron Progenitor Cells in Ambra1^+/−^ Female Embryos Are Vulnerable to Apoptosis

**Fig. 8:**
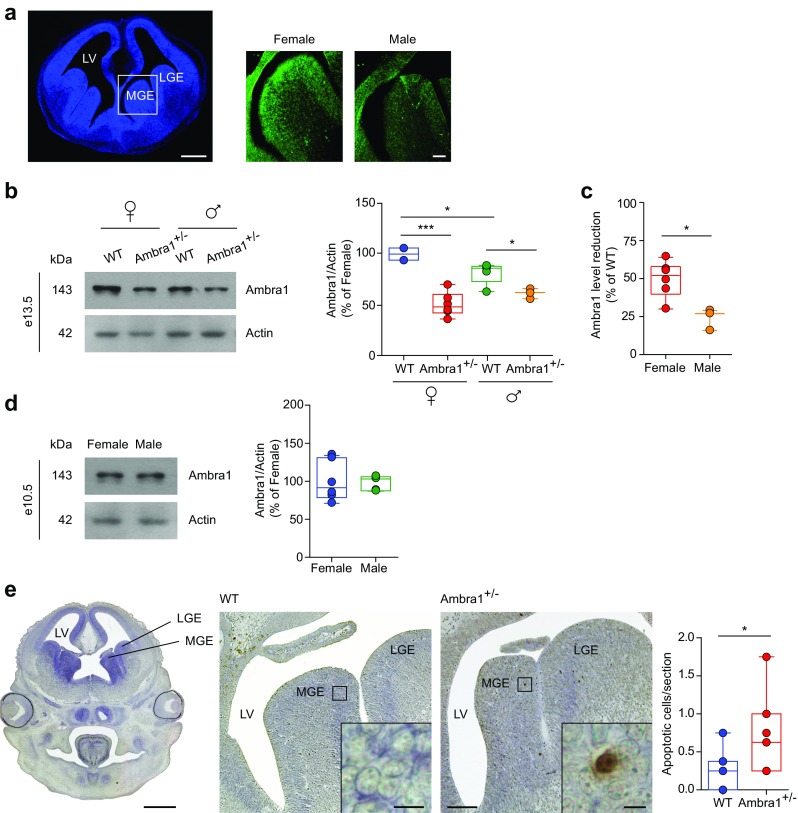
Female Ambra1^+/−^ e13.5 embryos show an increase of apoptotic cells in the MGE. **a** Left, DAPI (blue) stained coronal forebrain section from e13.5 WT female embryo; right, higher-magnification of the MGE showing labelling for Ambra1 protein (green) in female and male WT embryos. Ambra1 is expressed in the MGE and its expression is higher in female than in male embryos (scale bar: 500 μm; inset 50 μm). **b** Representative immunoblots of total Ambra1 protein extracted from the brain of female and male WT and Ambra1^+/−^ embryos at e13.5, probed with the Ambra1 antibody, and densitometric quantification of changes in gray values, expressed as % of female WT mice (females: *n* = 4 WT and 6 Ambra1^+/−^; males: *n* = 5 WT and 3 Ambra1^+/−^; two-way ANOVA for gender×genotype: *F*_1,14_ = 9.32, *P* = 0.009; Bonferroni’s post hoc test: WT female-Ambra1^+/−^ female, ***P = 1.00 × 10^−4^; WT female-WT male, *P < 0.05; WT male-Ambra1^+/−^ male, *P < 0.05). **c** Relative reduction from the respective WT of each gender, of Ambra1 in the brain of female and male Ambra1^+/−^ embryos at e13.5 (*n* as in **b**; two-tailed unpaired *t* test between genders: *P = 0.012). **d** Representative immunoblots of total Ambra1 protein from the brain of female and male WT embryos at e10.5, probed with the Ambra1 antibody, and densitometric quantification of changes in gray values (female: *n* = 6; male: *n* = 5). **e** Left, coronal forebrain section from e13.5 WT embryo; middle, higher-magnification images of the MGE and lateral ganglionic eminence (LGE) from a WT and Ambra1^+/−^ female embryo, with the indicated area enlarged in the inset to show a TUNEL-positive neuron in the Ambra1^+/−^ embryo (scale bars: 500, 50, and 5 μm, respectively). The plot shows the mean number of TUNEL-positive apoptotic neurons per section in the analyzed areas (*n* = 7 mice per genotype, four sections per animal; two-tailed unpaired *t* test *P = 0.044). LV, lateral ventricle

The large majority of mouse PV interneurons are generated in the MGE between e11 and e17, with a peak for CA1 at e13.5 [61, 62]. Using Ambra1 immunofluorescence, we confirmed that Ambra1 is expressed in the MGE of male and female e13.5 embryos, although the expression in males was less intense (Fig. 8a). Quantification of total protein levels showed that *Ambra1* heterozygosity reduces the protein expression of both female and male Ambra1^+/−^ embryos compared to WT siblings (Fig. 8b). In strict compliance with adult mice, Ambra1 expression in WT female embryos was significantly higher than WT males and the relative reduction of the protein in Ambra1^+/−^ embryos was more pronounced in females (Fig. 8b, c). The fact that Ambra1 expression is sexually dimorphic as early as e13.5 led us to investigate its expression in younger embryos, at an age preceding gonadic differentiation, to decipher whether the differential expression between genders is the result of sex steroid hormone influence or due to genetic factors [63]. Ambra1 expression levels in male and female WT embryos at e10.5 revealed no differences between genders (Fig. 8d), pointing to a different influence of sex steroid hormones in the protein expression in female compared to male mice.

Since Ambra1 is a pro-autophagic factor and autophagy is a self-degradative process promoting cell survival by elimination of damaged organelles and protein aggregates, we investigated whether the reduction of PV interneurons in Ambra1^+/−^ females was due to increased vulnerability of embryonic interneurons to cell death. Indeed, we found a higher number of apoptotic, TUNEL-positive cells in the MGE of e13.5 Ambra1^+/−^ female embryos compared with WT siblings (Fig. 8e), suggesting that Ambra1^+/−^ female interneurons are vulnerable to apoptosis, providing new insights relative to their genesis.

## Discussion

Growing experimental and clinical observations recognize that an altered interneuron development or function might impact on the ability to process complex information, by compromising the inhibitory/excitatory balance in the brain, a common feature in neurodevelopmental illnesses [10, 12–14]. Here, in a mouse suffering from haploinsufficiency for the *Ambra1* gene that develops a female-only phenotype characterized by socio-communicative deficits, we show that PV interneurons are reduced in the hippocampus, a brain area playing a crucial role in cognition and social behavior. Loss of PV interneurons is correlated with alterations in inhibitory synaptic transmission, CA3-CA1 synaptic plasticity, and pyramidal neuron spine density, as well as with reduced gamma oscillations and increased bicuculline-induced epileptiform activity, all pointing to an imbalance in the inhibition/excitation ratio in Ambra1^+/−^ female mice. The reduction of PV interneurons in Ambra1^+/−^ females appears to co-involve the loss of both dendritic- and perisomatic-targeting neurons (presumably bistratified and basket cells, respectively; [54]), the latter being evident in the presence of WIN55,212-2, but it does not appear to alter the hippocampal anatomical structure.

A striking outcome of this study is the fact that the described deficits are restricted to females. This is surprising considering that neurodevelopmental diseases such as ASDs and schizophrenia are diagnosed more frequently in males, although recent studies argue that many biases may influence this ratio, including an under-diagnosis in females [64]. A hint to the gender difference involving Ambra1 is the fact that WT females—both adults and e13.5 embryos—have higher levels of Ambra1 expression than males, suggesting that the stronger protein reduction in Ambra1^+/−^ mice affects females more. Moreover, our data from e10.5 embryos, showing no gender difference in Ambra1 levels, point to a causal role of sex steroid hormones in the differential expression of the protein between genders [63].

Which factors might underlie PV interneuron loss in Ambra1^+/−^ mice? The majority of hippocampal PV interneurons are generated from e11 to e17 in the MGE and migrate tangentially to reach the hippocampus [61, 62, 65]. Thus, the period between the second and third embryonic week is highly critical. Our evidence of increased TUNEL-positive apoptotic cells in the MGE of female Ambra1^+/−^ mice at e13.5, when most interneurons of the CA1 and CA3 are generated [61], strongly suggest that reduced Ambra1 levels confer increased susceptibility of Ambra1^+/−^ interneurons to apoptotic cell death. In fact, Ambra1 is pivotal for the elimination of damaged mitochondria [66], a form of autophagy known as mitophagy, making it likely that reduced Ambra1 levels could affect this process, shifting the balance toward apoptosis. This notion is particularly interesting considering that proper mitochondrial function is essential for interneuron development and migration [67], PV interneurons display high metabolic demands [68, 69], and mitochondrial dysfunction is linked to neurodevelopmental disorders, in which interneuron disturbances are also evident [14, 70].

Our work supports the notion that alterations in the function and/or number of PV interneurons, with the consequent changes in the brain inhibition/excitation ratio, might underlie behaviors that are reminiscent of neurodevelopmental disorders. We conclude that Ambra1 is differentially expressed between male and female brains following gonadal differentiation, and the haploinsufficiency condition is more damaging in female embryos, requiring a greater Ambra1 expression to complete physiological interneuron development.

## Electronic Supplementary Material

ESM 1 (PDF 376 kb)

## References

[1] Cheung C, Yu K, Fung G, Leung M, Wong C, Li Q, Sham P, Chua S, McAlonan G (2010). Autistic disorders and schizophrenia: related or remote? An anatomical likelihood estimation. PLoS One.

[2] Eack SM, Bahorik AL, McKnight SAF, Hogarty SS, Greenwald DP, Newhill CE, Phillips ML, Keshavan MS, Minshew NJ (2013). Commonalities in social and non-social cognitive impairments in adults with autism spectrum disorder and schizophrenia. Schizophr Res.

[3] Owen MJ, Sawa A, Mortensen PB (2016). Schizophrenia. Lancet Lond Engl.

[4] Regier DA, Kuhl EA, Kupfer DJ (2013). The DSM-5: classification and criteria changes. World Psychiatry Off J World Psychiatr Assoc WPA.

[5] St Pourcain B, Robinson EB, Anttila V, Sullivan BB, Maller J, Golding J, Skuse D, Ring S et al (2017) ASD and schizophrenia show distinct developmental profiles in common genetic overlap with population-based social communication difficulties. Mol Psychiatry. 10.1038/mp.2016.198 10.1038/mp.2016.198 PMC5382976 28044064

[6] Toal F, Bloemen OJN, Deeley Q, Tunstall N, Daly EM, Page L, Brammer MJ, Murphy KC, Murphy DGM (2009). Psychosis and autism: magnetic resonance imaging study of brain anatomy. Br J Psychiatry J Ment Sci.

[7] Schizophrenia Working Group of the Psychiatric Genomics Consortium (2014). Biological insights from 108 schizophrenia-associated genetic loci. Nature.

[8] Sahin M, Sur M (2015). Genes, circuits, and precision therapies for autism and related neurodevelopmental disorders. Science.

[9] de la Torre-Ubieta L, Won H, Stein JL, Geschwind DH (2016). Advancing the understanding of autism disease mechanisms through genetics. Nat Med.

[10] Rubenstein JLR, Merzenich MM (2003). Model of autism: increased ratio of excitation/inhibition in key neural systems. Genes Brain Behav.

[11] Gao R, Penzes P (2015). Common mechanisms of excitatory and inhibitory imbalance in schizophrenia and autism spectrum disorders. Curr Mol Med.

[12] Nelson SB, Valakh V (2015). Excitatory/inhibitory balance and circuit homeostasis in autism spectrum disorders. Neuron.

[13] Levitt P, Eagleson KL, Powell EM (2004). Regulation of neocortical interneuron development and the implications for neurodevelopmental disorders. Trends Neurosci.

[14] Marín O (2012). Interneuron dysfunction in psychiatric disorders. Nat Rev Neurosci.

[15] Le Magueresse C, Monyer H (2013). GABAergic interneurons shape the functional maturation of the cortex. Neuron.

[16] Hu H, Gan J, Jonas P (2014). Interneurons. Fast-spiking, parvalbumin^+^ GABAergic interneurons: from cellular design to microcircuit function. Science.

[17] Bartos M, Vida I, Jonas P (2007). Synaptic mechanisms of synchronized gamma oscillations in inhibitory interneuron networks. Nat Rev Neurosci.

[18] Fimia GM, Stoykova A, Romagnoli A (2007). Ambra1 regulates autophagy and development of the nervous system. Nature.

[19] Cecconi F, Di Bartolomeo S, Nardacci R (2007). A novel role for autophagy in neurodevelopment. Autophagy.

[20] Sepe S, Nardacci R, Fanelli F (2014). Expression of Ambra1 in mouse brain during physiological and Alzheimer type aging. Neurobiol Aging.

[21] Rietschel M, Mattheisen M, Degenhardt F (2012). Association between genetic variation in a region on chromosome 11 and schizophrenia in large samples from Europe. Mol Psychiatry.

[22] Heinrich A, Nees F, Lourdusamy A (2013). From gene to brain to behavior: schizophrenia-associated variation in AMBRA1 alters impulsivity-related traits. Eur J Neurosci.

[23] Dere E, Dahm L, Lu D, Hammerschmidt K, Ju A, Tantra M, KÃ¤stner A, Chowdhury K (2014). Heterozygous ambra1 deficiency in mice: a genetic trait with autism-like behavior restricted to the female gender. Front Behav Neurosci.

[24] Mitjans M, Begemann M, Ju A, Dere E, Wüstefeld L, Hofer S, Hassouna I, Balkenhol J (2017). Sexual dimorphism of AMBRA1-related autistic features in human and mouse. Transl Psychiatry.

[25] Scattoni ML, Ricceri L, Crawley JN (2011). Unusual repertoire of vocalizations in adult BTBR T+tf/J mice during three types of social encounters. Genes Brain Behav.

[26] Scattoni ML, Gandhy SU, Ricceri L, Crawley JN (2008). Unusual repertoire of vocalizations in the BTBR T+tf/J mouse model of autism. PLoS One.

[27] Maggio JC, Maggio JH, Whitney G (1983). Experience-based vocalization of male mice to female chemosignals. Physiol Behav.

[28] Whitney G, Coble JR, Stockton MD, Tilson EF (1973). Ultrasonic emissions: do they facilitate courtship of mice. J Comp Physiol Psychol.

[29] Whitney G, Nyby J (1979). Cues that elicit ultrasounds from adult male mice. Am Zool.

[30] Maggio JC, Whitney G (1985). Ultrasonic vocalizing by adult female mice (Mus musculus). J Comp Psychol Wash DC 1983.

[31] Moles A, D’amato F (2000). Ultrasonic vocalization by female mice in the presence of a conspecific carrying food cues. Anim Behav.

[32] Scattoni ML, Crawley J, Ricceri L (2009). Ultrasonic vocalizations: a tool for behavioural phenotyping of mouse models of neurodevelopmental disorders. Neurosci Biobehav Rev.

[33] Moy SS, Nadler JJ, Perez A, Barbaro RP, Johns JM, Magnuson TR, Piven J, Crawley JN (2004). Sociability and preference for social novelty in five inbred strains: an approach to assess autistic-like behavior in mice. Genes Brain Behav.

[34] Krashia P, Martini A, Nobili A (2017). On the properties of identified dopaminergic neurons in the mouse substantia nigra and ventral tegmental area. Eur J Neurosci.

[35] Paxinos G, Franklin KBJ Paxinos and Franklin’s the mouse brain in stereotaxic coordinates, 4th edn. Elsevier/Academic Press, Amsterdam

[36] Pagliarini V, Pelosi L, Bustamante MB (2015). SAM68 is a physiological regulator of SMN2 splicing in spinal muscular atrophy. J Cell Biol.

[37] Nobili A, Latagliata EC, Viscomi MT, Cavallucci V, Cutuli D, Giacovazzo G, Krashia P, Rizzo FR (2017). Dopamine neuronal loss contributes to memory and reward dysfunction in a model of Alzheimer’s disease. Nat Commun.

[38] Hartveit E, Veruki ML (2007). Studying properties of neurotransmitter receptors by non-stationary noise analysis of spontaneous postsynaptic currents and agonist-evoked responses in outside-out patches. Nat Protoc.

[39] Silverman JL, Yang M, Lord C, Crawley JN (2010). Behavioural phenotyping assays for mouse models of autism. Nat Rev Neurosci.

[40] Nguyen R, Morrissey MD, Mahadevan V, Cajanding JD, Woodin MA, Yeomans JS, Takehara-Nishiuchi K, Kim JC (2014). Parvalbumin and GAD65 interneuron inhibition in the ventral hippocampus induces distinct behavioral deficits relevant to schizophrenia. J Neurosci.

[41] Hashemi E, Ariza J, Rogers H, Noctor SC, Martínez-Cerdeño V (2017). The number of parvalbumin-expressing interneurons is decreased in the medial prefrontal cortex in autism. Cereb Cortex N Y N 1991.

[42] DeFelipe J (1997). Types of neurons, synaptic connections and chemical characteristics of cells immunoreactive for calbindin-D28K, parvalbumin and calretinin in the neocortex. J Chem Neuroanat.

[43] Leuba G, Saini K (1997). Colocalization of parvalbumin, calretinin and calbindin D-28k in human cortical and subcortical visual structures. J Chem Neuroanat.

[44] Buzsáki G, Draguhn A (2004). Neuronal oscillations in cortical networks. Science.

[45] Fuchs EC, Zivkovic AR, Cunningham MO, Middleton S, LeBeau FEN, Bannerman DM, Rozov A, Whittington MA (2007). Recruitment of parvalbumin-positive interneurons determines hippocampal function and associated behavior. Neuron.

[46] Tukker JJ, Fuentealba P, Hartwich K (2007). Cell type-specific tuning of hippocampal interneuron firing during gamma oscillations in vivo. J Neurosci.

[47] Cardin JA, Carlén M, Meletis K (2009). Driving fast-spiking cells induces gamma rhythm and controls sensory responses. Nature.

[48] Sohal VS, Zhang F, Yizhar O, Deisseroth K (2009). Parvalbumin neurons and gamma rhythms enhance cortical circuit performance. Nature.

[49] Lewis DA, Hashimoto T, Volk DW (2005). Cortical inhibitory neurons and schizophrenia. Nat Rev Neurosci.

[50] Orekhova EV, Stroganova TA, Nygren G, Tsetlin MM, Posikera IN, Gillberg C, Elam M (2007). Excess of high frequency electroencephalogram oscillations in boys with autism. Biol Psychiatry.

[51] Fisahn A, Pike FG, Buhl EH, Paulsen O (1998). Cholinergic induction of network oscillations at 40 Hz in the hippocampus in vitro. Nature.

[52] Gulyás AI, Szabó GG, Ulbert I (2010). Parvalbumin-containing fast-spiking basket cells generate the field potential oscillations induced by cholinergic receptor activation in the hippocampus. J Neurosci.

[53] Klausberger T, Somogyi P (2008). Neuronal diversity and temporal dynamics: the unity of hippocampal circuit operations. Science.

[54] Yi F, Ball J, Stoll KE (2014). Direct excitation of parvalbumin-positive interneurons by M1 muscarinic acetylcholine receptors: roles in cellular excitability, inhibitory transmission and cognition. J Physiol.

[55] Berghuis P, Dobszay MB, Ibanez RM, Ernfors P, Harkany T (2004). Turning the heterogeneous into homogeneous: studies on selectively isolated GABAergic interneuron subsets. Int J Dev Neurosci Off J Int Soc Dev Neurosci.

[56] Freund TF, Katona I (2007). Perisomatic inhibition. Neuron.

[57] Glickfeld LL, Atallah BV, Scanziani M (2008). Complementary modulation of somatic inhibition by opioids and cannabinoids. J Neurosci.

[58] Hutsler JJ, Zhang H (2010). Increased dendritic spine densities on cortical projection neurons in autism spectrum disorders. Brain Res.

[59] Penzes P, Cahill ME, Jones KA, VanLeeuwen JE, Woolfrey KM (2011). Dendritic spine pathology in neuropsychiatric disorders. Nat Neurosci.

[60] Tang G, Gudsnuk K, Kuo S-H (2014). Loss of mTOR-dependent macroautophagy causes autistic-like synaptic pruning deficits. Neuron.

[61] Danglot L, Triller A, Marty S (2006). The development of hippocampal interneurons in rodents. Hippocampus.

[62] Kelsom C, Lu W (2013). Development and specification of GABAergic cortical interneurons. Cell Biosci.

[63] Dewing P, Shi T, Horvath S, Vilain E (2003). Sexually dimorphic gene expression in mouse brain precedes gonadal differentiation. Brain Res Mol Brain Res.

[64] Halladay AK, Bishop S, Constantino JN (2015). Sex and gender differences in autism spectrum disorder: summarizing evidence gaps and identifying emerging areas of priority. Mol Autism.

[65] Pleasure SJ, Anderson S, Hevner R (2000). Cell migration from the ganglionic eminences is required for the development of hippocampal GABAergic interneurons. Neuron.

[66] Strappazzon F, Nazio F, Corrado M (2015). AMBRA1 is able to induce mitophagy via LC3 binding, regardless of PARKIN and p62/SQSTM1. Cell Death Differ.

[67] Lin-Hendel EG, McManus MJ, Wallace DC, Anderson SA, Golden JA (2016). Differential mitochondrial requirements for radially and non-radially migrating cortical neurons: implications for mitochondrial disorders. Cell Rep.

[68] Inan M, Zhao M, Manuszak M (2016). Energy deficit in parvalbumin neurons leads to circuit dysfunction, impaired sensory gating and social disability. Neurobiol Dis.

[69] Kann O (2016). The interneuron energy hypothesis: implications for brain disease. Neurobiol Dis.

[70] Rossignol DA, Frye RE (2012). Mitochondrial dysfunction in autism spectrum disorders: a systematic review and meta-analysis. Mol Psychiatry.

