# Supplementary material for: Ambra1 Shapes Hippocampal Inhibition/Excitation Balance: Role in Neurodevelopmental Disorders
Source: Mol Neurobiol. 2018 Feb 27;55(10):7921–40. doi: 10.1007/s12035-018-0911-5 (PMC6132777; doi:10.1007/s12035-018-0911-5)
Supplement: Supplementary file 1 — (PDF 376 kb) [file 12035_2018_911_MOESM1_ESM.pdf]

SUPPLEMENTARY INFORMATION

Ambra1 shapes hippocampal inhibition/excitation balance: role in neurodevelopmental disorders

Molecular Neurobiology

Annalisa Nobili[#], Paraskevi Krashia[#], Alberto Cordella, Maria Concetta Dell'Acqua, Angela Caruso, Annabella Pignataro, Ramona Marino, Francesca Sciarra, Livia La Barbera, Filippo Biamonte, Maria Luisa Scattoni, Martine Ammassari-Teule, Francesco Cecconi, Nicola Berretta, Flavio Keller, Nicola Biagio Mercuri, Marcello D'Amelio[*]

Contact Information:

[*]Corresponding author: m.damelio@unicampus.it

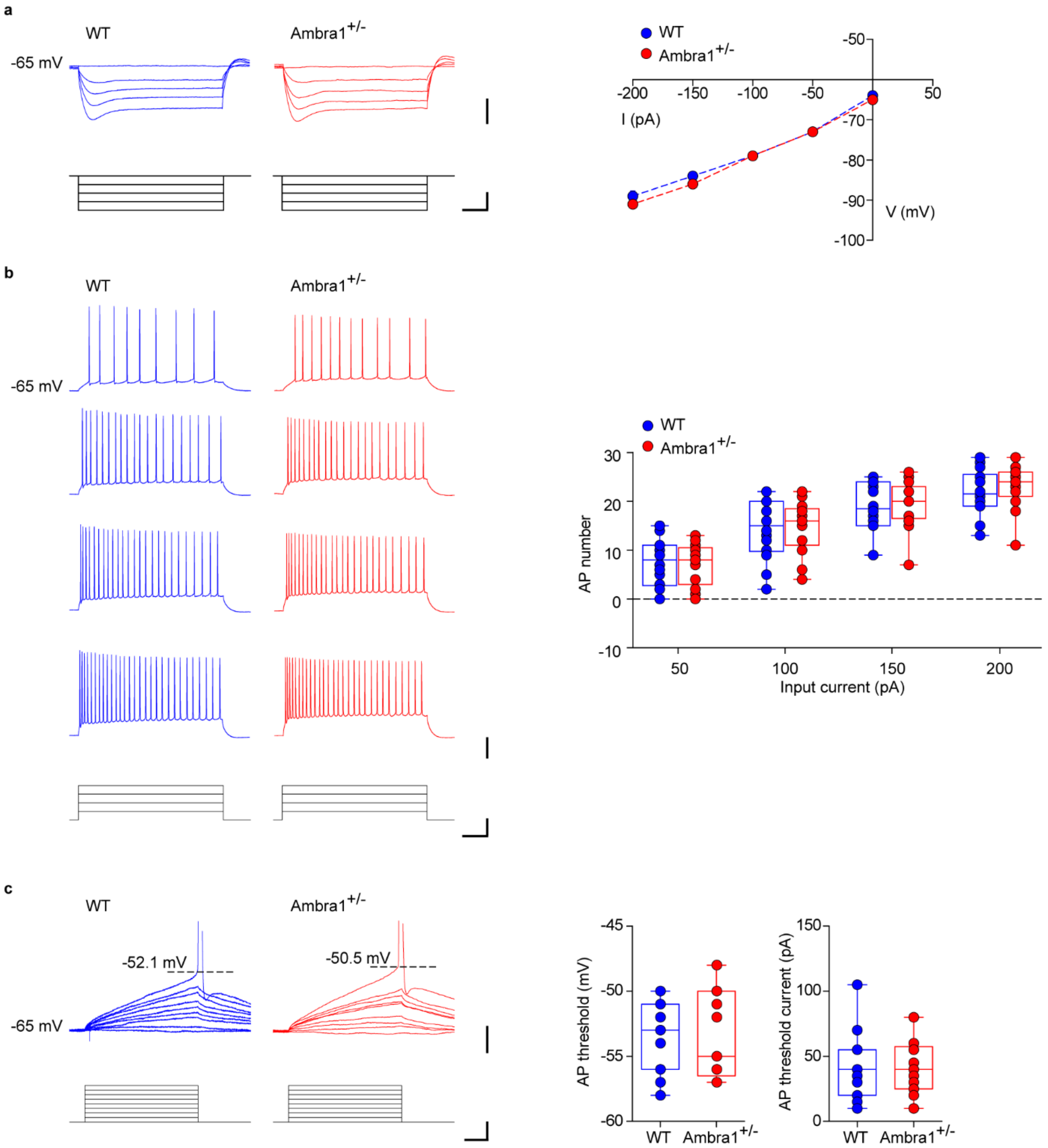

Suppl Fig 1: The excitability of CA1 pyramidal neurons in not altered in Ambra1$^{+/-}$ female mice

(**A**) Representative sub-threshold responses (at 50 pA stepped current injections; 600 ms protocols are shown below; scale bars: 100 ms, 15 mV top, 100 pA bottom) from a female WT and Ambra1$^{+/-}$ CA1 pyramidal neuron and mean I/V plots (± sem). The mean R_{in} does not differ between WT and Ambra1$^{+/-}$ mice (n = 15 WT and 13 Ambra1$^{+/-}$ neurons; 3 WT and 4 Ambra1$^{+/-}$ mice). (**B**) Example action potential (AP) firing patterns from the same neurons as in (**A**), in response to 50 pA-stepped

depolarizing current injections (50, 100, 150 and 200 pA current injection, from top to bottom; scale bars: 100 ms, 25 mV top, 100 pA bottom). The AP number at different input currents does not differ between WT and Ambra1$^{+/-}$ mice (n = 15 WT and 13 Ambra1$^{+/-}$ neurons; 3 WT and 4 Ambra1$^{+/-}$ mice). (**C**) Example current-clamp recordings (scaled to show the AP threshold) and respective drive current (5 pA stepped current injections; 50 ms duration; scale bars: 10 ms, 5 mV top, 20 pA bottom) to induce AP firing in the CA1 pyramidal neurons shown in **A** and **B**. The AP threshold and drive current do not differ between WT and Ambra1$^{+/-}$ neurons (n = 15 WT and 13 Ambra1$^{+/-}$ neurons; 3 WT and 4 Ambra1$^{+/-}$ mice).

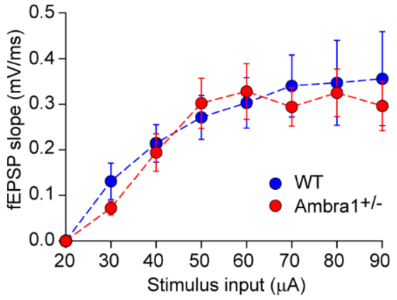

Suppl Fig 2: Input/output curves of CA3-to-CA1 fEPSP slope in female mice

Input/output curves of CA3-to-CA1 fEPSP mean slope (± sem) at stimulations of increasing intensity, showing no difference in basal synaptic transmission between WT and Ambra1$^{+/-}$ female mice (n = 19 WT and 25 Ambra1$^{+/-}$ slices from 6 mice per genotype).

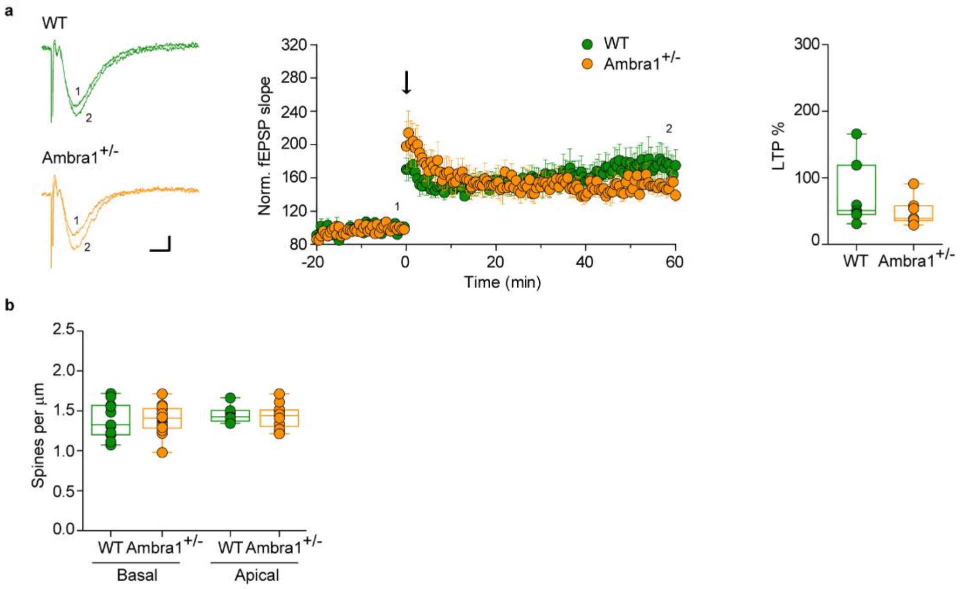

Suppl Fig 3: The CA3-to-CA1 synaptic plasticity and spine density are unchanged in male Ambra1$^{+/-}$ mice

(**A**) Running plots of normalized CA3-to-CA1 fEPSP mean slope (± sem) recorded from the dendritic region of CA1 pyramidal neurons in hippocampal slices from male WT and Ambra1$^{+/-}$ mice. The arrow indicates the time when a high frequency conditioning train was delivered on the Schaffer collaterals. The traces (scale bars: 5 ms, 0.1 mV) are superimposed fEPSPs recorded during baseline (1) and 1 h after LTP induction (2). The box-and-whisker plot indicates the degree of potentiation, measured as fEPSP slope increase from baseline, 55-60 min after the train ($n = 7$ slices from 3 mice per genotype). (**B**) Spine density of basal and apical dendrites of CA1 pyramidal neurons in WT and Ambra1$^{+/-}$ male mice, expressed as spine number per 1 µm dendrite segment.

Suppl Table 1: Summary of sub-threshold and supra-threshold membrane properties of CA1 pyramidal neurons.

R_{in}: input resistance; V_{rest}: resting membrane potential; AP: action potential; FWHM: full-width at half-maximum amplitude of action potential

	**WT (n = 15)**	**Ambra1$^{+/-}$ (n = 13)**	**Unpaired *t*-test *P* value**
**Sub-threshold**			
R_{in} (MΩ)	122.94 ± 6.76	128.88 ± 3.84	0.454
V_{rest} (mV)	-63.73 ± 1.13	-62.54 ± 0.58	0.359
Sag ratio	0.930 ± 0.02	0.928 ± 0.02	0.838
**Supra-threshold**			
AP Threshold (mV)	-53.50 ± 0.64	-53.63 ± 0.95	0.912
AP Amplitude (mV)	90.58 ± 3.57	92.54 ± 3.29	0.691
FWHM (msec)	1.19 ± 0.04	1.19 ± 0.05	0.910
AP Rise slope (dV/dt)	200.70 ± 17.10	209.28 ± 14.44	0.705
AP Decay slope (dV/dt)	62.30 ± 5.55	59.15 ± 5.26	0.684
